# A SAM oligomerization domain shapes the genomic binding landscape of the LEAFY transcription factor

**DOI:** 10.1038/ncomms11222

**Published:** 2016-04-21

**Authors:** Camille Sayou, Max H. Nanao, Marc Jamin, David Posé, Emmanuel Thévenon, Laura Grégoire, Gabrielle Tichtinsky, Grégoire Denay, Felix Ott, Marta Peirats Llobet, Markus Schmid, Renaud Dumas, François Parcy

**Affiliations:** 1 Laboratoire de Physiologie Cellulaire et Végétale, Université Grenoble Alpes, CNRS UMR5168, CEA/DRF/BIG, INRA UMR 1417, 17, avenue des Martyrs, 38054 Grenoble, France; 2 European Molecular Biology Laboratory, 71, avenue des Martyrs, 38042 Grenoble, France; 3 Institut de Biologie Structurale CEA/DRF, CNRS, Université Grenoble Alpes, 71, avenue des Martyrs, 38044 Grenoble, France; 4 Department of Molecular Biology, Max Planck Institute for Developmental Biology, 72076 Tübingen, Germany; 5 Instituto de Hortofruticultura Subtropical y Mediterránea, Universidad de Málaga-Consejo Superior de Investigaciones Científicas, Departamento de Biología Molecular y Bioquímica, Facultad de Ciencias, Universidad de Málaga, 29071 Málaga, Spain; 6 Umeå Plant Science Centre, Department of Plant Physiology, Umeå University, SE-901 87 Umeå, Sweden; 7 Centre for Molecular Medicine and Therapeutics, Child and Family Research Institute, University of British Columbia, Vancouver, British Columbia, Canada V5Z 4H4

## Abstract

Deciphering the mechanisms directing transcription factors (TFs) to specific genome regions is essential to understand and predict transcriptional regulation. TFs recognize short DNA motifs primarily through their DNA-binding domain. Some TFs also possess an oligomerization domain suspected to potentiate DNA binding but for which the genome-wide influence remains poorly understood. Here we focus on the LEAFY transcription factor, a master regulator of flower development in angiosperms. We have determined the crystal structure of its conserved amino-terminal domain, revealing an unanticipated Sterile Alpha Motif oligomerization domain. We show that this domain is essential to LEAFY floral function. Moreover, combined biochemical and genome-wide assays suggest that oligomerization is required for LEAFY to access regions with low-affinity binding sites or closed chromatin. This finding shows that domains that do not directly contact DNA can nevertheless have a profound impact on the DNA binding landscape of a TF.

## Abstract (web summary)

The LEAFY transcription factor is a master regulator of flower development in plants. Here the authors describe the structure of a LEAFY oligomerization domain and show that mutations that disrupt oligomerization alter its capacity to bind low affinity and poorly accessible target sites.

## Graphical abstract

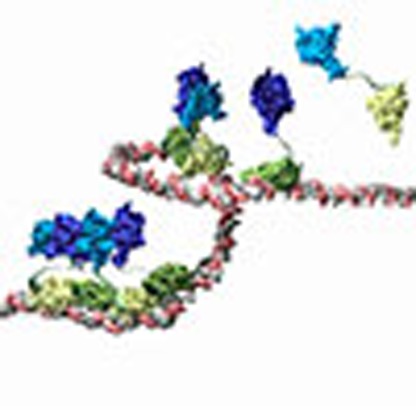

Transcription factors (TFs) play key roles during development and differentiation^1^. By binding to short stretches of DNA called *cis*-elements or TF binding sites (TFBS) and thereby regulating gene expression, they are able to decode the regulatory information present in genomes^2^. Based on structural studies and genome-wide analyses, the rules governing TF DNA interactions are becoming clearer and DNA-binding models with increased prediction power are available^3^,^4^. TF DNA-binding specificity is primarily determined by their DNA-binding domains (DBDs) through contact with bases and DNA shape readout^4^. DBD are often dimeric or sometimes tetrameric, thereby increasing the TFBS length and the TF specificity^3^. In addition to their DBD, some TF also possess an oligomerization domain^5^. Such a feature is found in several types of factors such as members of the E26 transformation-specific or E-twenty-six (ETS) family^6^, auxin response factors^7^, some zinc-finger TFs^8^ or the PRH/Hex^9^ TF. How such domains modify the repertoire of TF target regions at the genome-wide scale is largely unknown.

Here we focus on LEAFY (LFY), a plant-specific TF essential for flower development^10^. LFY is one of the few master regulators of flower development, as it integrates environmental and endogenous signals to orchestrate the whole floral network^11^,^12^,^13^,^14^. It first triggers the early emergence of a bulge of stem cells on the flanks of the shoot apex^15^,^16^ and subsequently specifies their floral identity by directly inducing floral homeotic genes such as *APETALA1* (*AP1*), *APETALA3* (*AP3*) or *AGAMOUS* (*AG*) and by repressing the shoot meristem identity gene *TERMINAL FLOWER1* (*TFL1*)^10^,^11^. Comprehensive lists of genes bound and possibly regulated by LFY have been established by combining genome-wide chromatin immunoprecipitation (ChIP) experiments and transcriptomic approaches^17^,^18^.

The function of LFY as a master regulator of floral development is most obvious when expressed from a constitutive promoter: it is sufficient to trigger early flowering and ectopic flower production^19^. Together with co-regulators such as the homeodomain TF WUSCHEL or the F-Box protein UNUSUAL FLORAL ORGANS, it can even induce flowers from root or leaf tissue, respectively^20^,^21^. This acquisition of floral fate is remarkable, as several LFY targets including *AG* and *AP3* are known to be under the repression of two Polycomb repressive complexes (PRC1 and PRC2) (refs 22, 23), which ectopic LFY expression is apparently sufficient to override.

Structure–function studies have demonstrated that LFY binds semi-palindromic 19-bp DNA elements through its highly conserved C-terminal DBD, a unique helix-turn-helix fold that by itself dimerizes on DNA (Fig. 1a)^17^,^18^,^24^,^25^. A biophysical model describing LFY DNA-binding specificity *in vitro* was built and accurately predicts LFY-binding sites (LFYbs) in the *Arabidopsis thaliana* genome^17^,^26^. In addition to its well-characterized DBD, LFY possesses a second conserved domain at its amino terminus (LFY-N) (Fig. 1a). This domain has been proposed to mediate LFY dimerization by forming a leucine zipper^27^. Here we report the crystallographic structure of LFY-N and elucidate its function using a combination of biochemical and genomic approaches. Our results demonstrate that LFY-N is a Sterile Alpha Motif (SAM) domain that mediates LFY oligomerization. It allows LFY to bind to regions lacking high-affinity LFYbs and confers on LFY the ability to access closed chromatin regions.

## Results

### LFY N-terminal domain is a SAM

**Figure 1:**
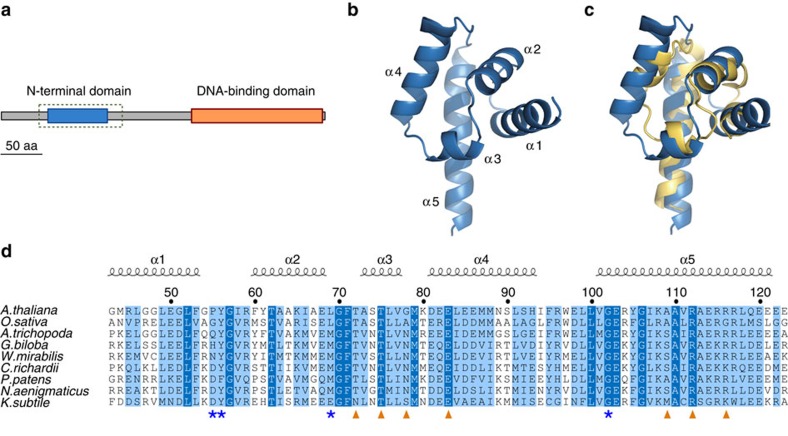
LFY N-terminus is a SAM domain. (**a**) Domain representation of GbLFY protein with the N-terminal domain in blue and DBD in orange. Dashes indicate the region used to obtain the crystal structure. Scale bar, 50 amino acids (aa). (**b**) Crystal structure of GbLFY-N monomer. α, α-helices. (**c**) Superimposition of GbLFY-N (blue) and the SAM domain of p63α (yellow, PDB 2Y9U)^66^. The two structures are very similar and can be superimposed with a root mean square deviation of 1.9 Å for 62 Cα. (**d**) Sequence alignment of LFY-N domain. The residue numbering refers to *A. thaliana* LFY sequence; the secondary structure from GbLFY-SAM crystal structure is indicated above the alignment; orange triangles and blue stars indicate residues involved in the interaction between SAM monomers through the lateral and main chain, respectively.

**Figure 2:**
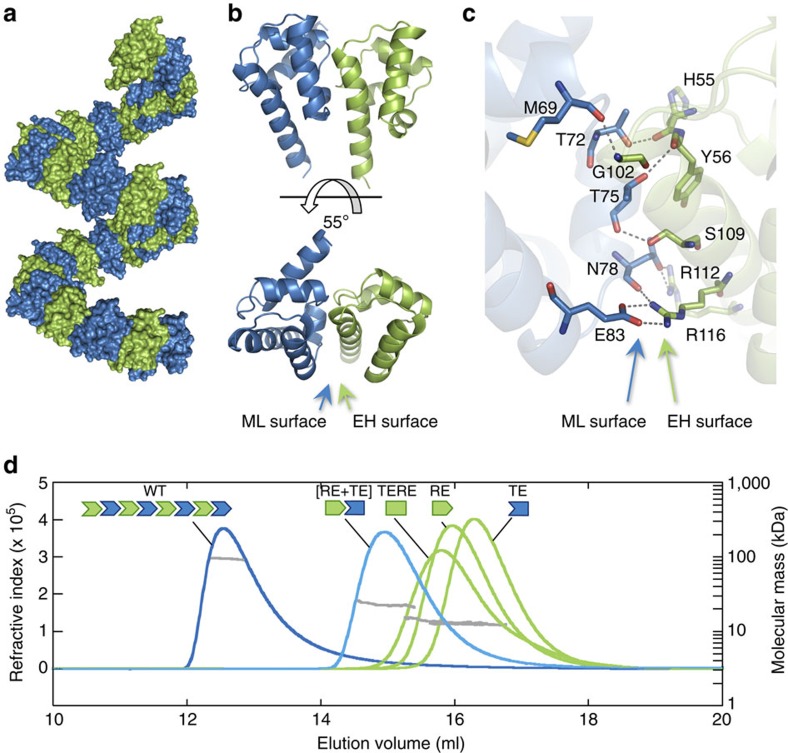
LFY-SAM is an oligomerization domain. (**a**) Helical polymer of GbLFY-SAM in the crystal structure. Twenty-eight monomers are shown, alternatively coloured in blue and green. (**b**) Side and top views (upper and bottom panels, respectively) of the GbLFY-SAM dimer in the asymmetric unit of the crystal. ML and EH interaction surfaces are indicated. (**c**) Detailed view of GbLFY-SAM oligomerization interface. Residues mediating contacts between the two monomers are shown as sticks; residues on the ML and EH surfaces are in blue and green, respectively. (**d**) SEC-MALLS analysis of GbLFY-SAM (WT), GbLFY-SAM_TE_ (TE, T75E substitution at the ML surface) and GbLFY-SAM_RE_ (RE, R112E substitution at the EH surface), GbLFY-SAM_TERE_ (TERE, T75E, R112E) double mutant and GbLFY-SAM_[RE+TE]_ ([RE+TE], an equimolar mixture of GbLFY-SAM_TE_ and GbLFY-SAM_RE_). 50 μl at ∼4.5 mg ml^−1^ of proteins were used. Elution profiles were monitored by excess refractive index (left ordinate axis). The grey line under each elution peak shows the molecular mass distribution (right ordinate axis). Measured molecular masses (reported in Supplementary Table 1) show that GbLFY-SAM is oligomeric; GbLFY-SAM_TE_, GbLFY-SAM_RE_ and GbLFY-SAM_TERE_ are monomeric; and GbLFY-SAM_[RE+TE]_ is dimeric.

We used a structural approach to unravel the function of the N-terminal domain of LFY. After heterologously overexpressing LFY-N domains from different plant species, we were able to determine the crystal structure for GbLFY-N from the gymnosperm *Ginkgo biloba* at 2.3 Å resolution (Table 1 and Supplementary Fig. 1). This structure showed that the GbLFY-N monomer is made up of five α-helices separated by four loops (Fig. 1b). Comparison against the Protein Data Bank revealed a strong structural similarity with the SAM, also called Pointed domain^28^ (Fig. 1c), a resemblance that could not be predicted from LFY-N sequences. SAM domains have not been characterized in plants, but they are common protein motifs in other eukaryotes. They interact with proteins, DNA, RNA and lipids, and are involved in many cellular functions including transcriptional regulation and signal transduction^6^,^28^. Some SAM domains are able to self-associate as oligomers through the so-called mid-loop (ML) and end-helix (EH) surfaces^29^,^30^. GbLFY-SAM appears to belong to this class: in the crystal, it forms a head-to-tail polymer (Fig. 2a) with monomers contacting their neighbours through two polar surfaces (Fig. 2b). The ML surface comprises negative and polar residues (Fig. 2c), including T72, T75 and E83, which interact with the positively charged EH surface through hydrogen bonds and salt bridges, in particular via R112 and R116 (residue numbering refers to LFY from *A. thaliana*—AtLFY—sequence; Fig. 1d).

### LFY-SAM is an oligomerization domain

To validate the interactions observed in the crystal in solution, we analysed the oligomerization state of the wild-type (WT) GbLFY-SAM protein and of mutants at the ML surface (T75E substitution, GbLFY-SAM_TE_), EH surface (R112E substitution, GbLFY-SAM_RE_) or at both surfaces (GbLFY-SAM_TERE_) (Fig. 2d). Size-exclusion chromatography coupled to multi-angle laser light scattering (SEC-MALLS) showed that the WT GbLFY-SAM domain formed oligomers of variable size in solution, depending on protein concentration, containing up to eight monomers (Fig. 2d, Supplementary Fig. 2 and Supplementary Table 1). In comparison, all mutations affecting the interaction interface completely abolished LFY oligomerization and GbLFY-SAM_TE_, GbLFY-SAM_RE_ and GbLFY-SAM_TERE_ proteins were found to be exclusively monomeric (Fig. 2d and Supplementary Table 1). However, when mixed at an equimolar ratio, the GbLFY-SAM_TE_ and GbLFY-SAM_RE_ single-face mutants retained the ability to interact, forming a GbLFY-SAM_[RE+TE]_ dimer (Fig. 2d and Supplementary Table 1), thus showing that the different mutations did not alter the overall fold of the SAM domain, and that single face mutant proteins still displayed one functional interaction surface. These results further showed that the head-to-tail arrangement observed in the crystal structure reflects the state of LFY-SAM in solution, which forms an oligomer of limited size in the conditions of this study. The point mutations we designed allowed us to control the oligomerization state, creating either monomeric or dimeric variants of LFY-SAM instead of higher-order oligomers.

Comparing LFY sequences from multiple species throughout the plant kingdom revealed that the key interaction residues of the SAM domain are well conserved (Fig. 1d and Supplementary Fig. 2d), suggesting that the capacity to oligomerize is a general characteristic of LFY-SAM in all species from algae to angiosperms. This hypothesis is supported by the behaviour of the SAM domain of *A. thaliana* LFY (AtLFY-SAM) whose molecular mass in solution increased with its concentration, similar to what was observed for GbLFY-SAM (Supplementary Fig. 2).

### LFY-SAM_TERE_ mutants have highly reduced function in planta

Several mutations that compromise LFY function in rice and *A*. *thaliana* map to the SAM domain^27^,^31^. However, according to our structural data, these positions correspond to buried hydrophobic residues that are unlikely to directly affect oligomerization but are more likely to be important for proper domain folding. Only the very weak *lfy-22* allele in *A. thaliana*^32^ carries a mutation (G70D) that localizes to the SAM ML surface and possibly slightly weakens oligomerization. Therefore, to specifically assess the importance of oligomerization for LFY function, we tested whether the TERE mutation affects AtLFY ability to control flower development in the genetically amenable *A. thaliana* plant.

**Figure 3:**
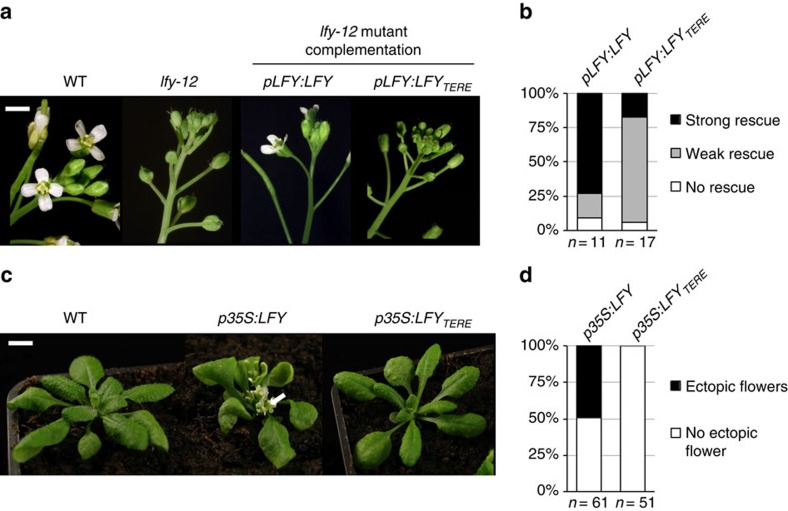
Mutations to the LFY-SAM oligomerization domain impair LFY floral function in *A. thaliana.* (**a**) Pictures of WT, *lfy-12* mutant and *lfy-12* expressing *pLFY:LFY* or *pLFY:LFY*_*TERE*_ inflorescences from 40-day-old plants grown under long day conditions (scale bar, 2 mm). (**b**) Distribution of *pLFY:LFY* and *pLFY:LFY*_*TERE*_ primary transformants in phenotypic complementation classes (see Supplementary Table 2 for details). (**c**) Pictures of WT and transgenic plants expressing *p35S:LFY* or *p35S:LFY*_*TERE*_ grown for 30 days under long-day conditions (scale bar, 1 cm). The white arrow indicates ectopic rosette flowers. (**d**) Proportion of the *p35S:LFY* and *p35S:LFY*_*TERE*_ primary transformants showing ectopic rosette flowers.

We first tested whether AtLFY_TERE_ could complement the *lfy-12*-null mutant, in which flowers are replaced by shoots or sterile shoot/flower intermediate structures lacking petals and stamens (Fig. 3a)^33^. When expressed under the control of the *LFY* endogenous promoter (*pLFY*), AtLFY_TERE_ complemented the *lfy-12* mutant much less efficiently than AtLFY (Fig. 3a,b and Supplementary Table 2, Mann–Whitney rank test, *P*=0.01006). Whereas most of *pLFY:LFY lfy-12* primary transformants produced fertile flowers, the majority of *pLFY:LFY*_*TERE*_ *lfy-12* primary transformants produced only a few fertile flowers at the base of the inflorescence. These results indicated that LFY activity was reduced, although not entirely abolished, when its oligomerization was compromised.

To further assess the importance of the SAM domain for LFY master regulator function, we expressed *AtLFY* from the *p35S* constitutive promoter and monitored its effect at an early developmental stage, when other pathways contributing to flower development have only a minimal impact. When expressed constitutively, AtLFY is known to induce early flowering, and to trigger the precocious termination of the shoots and the formation of ectopic flowers from the axils of rosette leaves^19^. About half of the 61 *p35S:LFY* T1 plants we generated showed this characteristic phenotype. In contrast, ectopic flowers were never observed in 51 *p35S:LFY*_*TERE*_ T1 transformants (Fig. 3c,d, Fisher's exact test, *P*-value=1.5 × 10^−10^) despite the fact that AtLFY and AtLFY_TERE_ proteins were expressed at similar levels (Supplementary Fig. 3). Taken together, these results provide evidence that LFY-SAM domain oligomerization is required for the function of LFY as a master switch in flower initiation.

### SAM_TERE_ mutations do not alter single LFYbs binding *in vitro*

Next, we aimed to pinpoint how a functional SAM domain contributes to LFY function at the molecular level. As a TF, LFY specifically binds to *cis*-elements present in the regulatory regions of its target genes. Hence, we tested whether LFY mutants with altered oligomerization status differed in DNA-binding ability. For other SAM proteins, such analyses were hampered by the difficulty in producing recombinant soluble proteins due to the aggregation and precipitation triggered by the SAM domain^34^. By optimizing the purification protocol, we successfully produced a near full-length GbLFY protein (GbLFYΔ) lacking only the 53 non-conserved N-terminal amino acids preceding the SAM domain.

**Figure 4:**
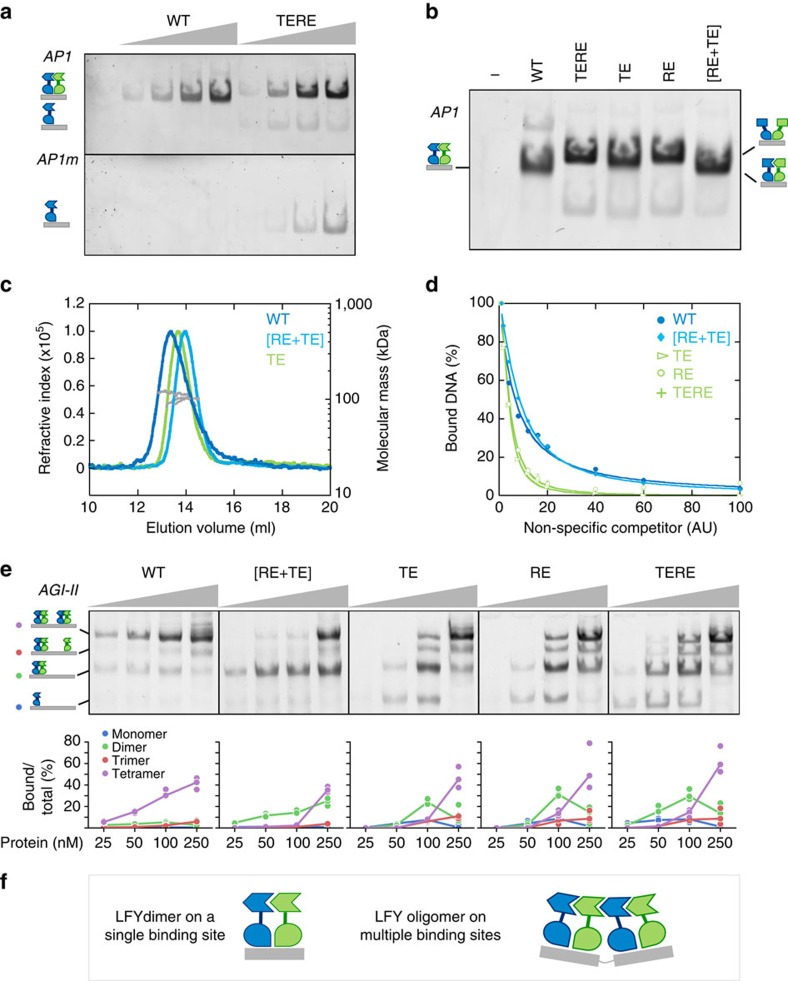
Role of the LFY-SAM oligomerization domain in *in vitro* DNA binding. (**a**) EMSA with 10 nM *AP1* or *AP1m* DNA probe and 0, 50, 100, 250 and 500 nM GbLFYΔ (WT) or GbLFYΔ_TERE_ (TERE) protein. This experiment was performed four times with similar results. (**b**) EMSA with 10 nM *AP1* DNA and 500 nM GbLFYΔ (WT), GbLFYΔ_[RE+TE]_ [RE+TE], GbLFYΔ_TE_ (TE), GbLFYΔ_RE_ (RE) or GbLFYΔ_TERE_ (TERE) protein. This experiment was performed twice with similar results. (**c**) SEC-MALLS molecular mass analysis of GbLFYΔ, GbLFYΔ_TE_ and GbLFYΔ_[RE+TE]_ in complex with the *AP1* probe. Fifty microlitres containing 2.6 mg ml^−1^ (60 μM) protein and 15 μM DNA were used. Elution profiles were monitored by excess refractive index (left ordinate axis). The line under each elution peak shows the molecular mass distribution (right ordinate axis). The measured molecular mass for each complex is reported in Supplementary Table 1 and is consistent with that of a protein dimer on DNA. The SEC experiment was performed twice and the MALLS measurement was performed once. (**d**) Percentage of DNA bound to LFY as a function of nonspecific unlabelled competitor DNA concentration. Quantifications were based on EMSA shown in Supplementary Fig. 4c. This experiment was performed four times with similar results. (**e**) EMSA with 10 nM *AGI-II* DNA probe and 25, 50, 100 and 250 nM GbLFYΔ, GbLFYΔ_[RE+TE]_, GbLFYΔ_TE_, GbLFYΔ_RE_ or GbLFYΔ_TERE_ protein. Quantifications show the percentage of total DNA bound by one (monomeric complex, blue), two (dimeric complex, green), three (trimeric complex, red) or four (tetrameric complex, violet) LFY proteins. EMSA have been performed in triplicate and the fit follows the median value. Formation of the tetrameric complex starts at lower concentrations for WT GbLFY as compared with the mutant GbLFY versions. (**f**) Schematic representation of LFY bound as dimer on a single binding site (left) and as tetramer on two binding sites (right) deduced from the EMSA and SEC-MALLS analysis. DNA is in grey and LFY in blue and green. For all EMSAs, only the protein–DNA complexes are shown.

As GbLFY and AtLFY have comparable DNA-binding specificities^24^, we tested GbLFYΔ binding to a DNA fragment carrying a single high-affinity AtLFYbs (*AP1* probe) from the promoter of the *A. thaliana AP1* gene^35^. Electrophoretic mobility shift assay (EMSA) and SEC-MALLS experiments showed that GbLFYΔ and GbLFYΔ_TERE_ bind *AP1* DNA mostly as dimers (Fig. 4c and Supplementary Table 1) and with the same apparent affinity (Fig. 4a). Thus, the SAM domain is not essential for the formation of the LFY dimer/DNA complex. This is consistent with previous studies showing that LFY-DBD by itself dimerizes on DNA^25^.

We noticed, however, a slight migration shift for GbLFYΔ_TE_, GbLFYΔ_RE_ and GbLFYΔ_TERE_ complexes when compared with GbLFYΔ in EMSA (Fig. 4a,b). This shift disappeared when GbLFYΔ_RE_ and GbLFYΔ_TE_ (hereafter called GbLFYΔ_[RE+TE]_) were mixed at equimolar concentration (Fig. 4b). This difference in migration behaviour might be due to a slightly different conformation of the complexes when the SAM domain is monomeric. A comparable result was obtained for the AtLFY protein (Supplementary Fig. 4a). Taken together, these findings indicate that LFY-SAM oligomerization is not required to trigger dimer formation on DNA but it appears to only slightly influence the migration of the LFY/DNA complex.

### The SAM domain prevents LFY DNA binding as monomer

In addition to the main dimeric protein/DNA complex, GbLFYΔ_TE_, GbLFYΔ_RE_ and GbLFYΔ_TERE_ protein variants showed a weak monomeric complex with the *AP1* probe in EMSA (Fig. 4b). This was particularly obvious when using a mutated probe, *AP1m*, which contained mutations on each side of the LFYbs pseudo-palindrome and was not bound by WT GbLFYΔ (Fig. 4a bottom panel). GbLFYΔ_TERE_ was also able to bind to DNA probes that carried mutations in either half of the palindromic binding site (*AP1m1* and *AP1m2* probes) as a monomer, whereas GbLFYΔ showed very little binding to such probes (Supplementary Fig. 4b). These experiments suggest that the LFY-SAM domain might prevent monomeric LFY binding on the numerous half sites present in a genome. To further investigate this possibility, we performed competition EMSA with increasing amounts of nonspecific unlabelled DNA competitor (fish genomic DNA). We found that GbLFYΔ_TE_, GbLFYΔ_RE_ and GbLFYΔ_TERE_ dissociated from *AP1* at lower competitor concentrations than GbLFYΔ and GbLFYΔ_[RE+TE]_ (Fig. 4d and Supplementary Fig. 4c). Thus, the SAM domain appears to favour LFY dimeric binding at the expense of a less specific monomeric interaction with DNA.

### LFY-SAM_TERE_ mutants show impaired cooperative DNA binding

Because of their ability to oligomerize, SAM domains have been proposed to allow cooperative DNA binding on regions with multiple TFBS^36^. This property has been inferred from SAM protein variants whose oligomerization potential was limited to dimerization^34^ but never established for a WT SAM protein. To test whether LFY-SAM could affect binding on DNA fragments with multiple binding sites *in vitro*, we used probes carrying two nearby LFYbs, either synthetic or from the *AG* regulatory region (*AGI-II*)^17^. With these probes, the GbLFYΔ protein formed one main complex corresponding to two bound dimers (Fig. 4e and Supplementary Figs 4d and 5). At low concentrations, GbLFYΔ_[RE+TE]_, which mimics a WT dimer unable to further oligomerize (Fig. 2d), was not as efficient as GbLFYΔ at forming a tetrameric complex and the tetramer/dimer ratio was lower for GbLFYΔ_[RE+TE]_ than for GbLFYΔ (Fig. 4e and Supplementary Fig. 5). As expected, all monomeric mutants (GbLFYΔ_TE_, GbLFYΔ_RE_ and GbLFYΔ_TERE_) were also impaired in tetramer formation at low concentrations (Fig. 4e and Supplementary Fig. 5). In these assays, the AtLFY protein behaved similarly as GbLFYΔ (Supplementary Fig. 4a), indicating that mediating cooperative binding to multiple LFYbs is an intrinsic property of the LFY-SAM domain and is important for the formation of tetrameric complexes (Fig. 4f).

### A functional SAM domain is required for DNA binding *in vivo*

**Figure 5:**
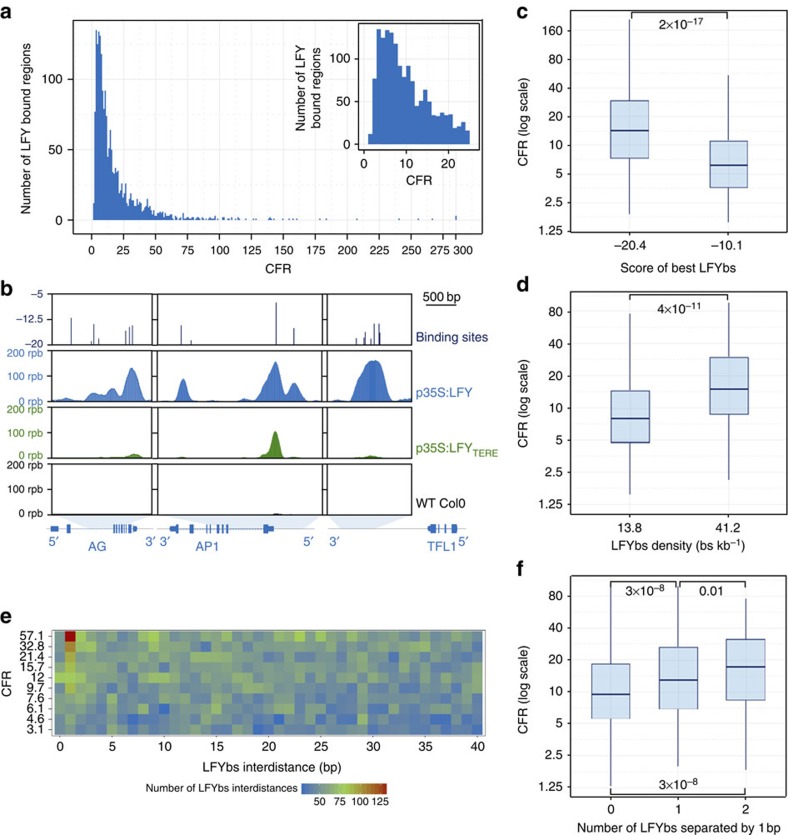
Mutations altering LFY oligomerization affect genome-wide LFY DNA binding *in planta.* (**a**) Histogram showing the distribution of CFR values for the LFY-bound regions in *p35S:LFY*. The inset shows an enlargement for low CFR. (**b**) AtLFY and AtLFY_TERE_ binding profiles on the *AG* (left), *AP1* (middle) and the *TFL1* (right) genomic regions. ChIP-Seq read coverage is shown for *p35S:LFY* (blue), *p35S:LFY*_*TERE*_ (green) and Col-0 (black, background signal). The upper panel shows the LFYbs locations and scores. Blue boxes at the bottom indicate exons. rpb, read per base. (**c**) Boxplot showing the CFR of the LFY-bound regions with LFYbs of lowest and highest score (first and last deciles). The *x* axis shows the median LFYbs best score value. *P*-value for Mann–Whitney rank test is indicated on the graph. (**d**) Boxplot showing the CFR of the LFY-bound regions with lower and higher LFYbs density (first and last deciles of LFY-bound regions). The *x* axis shows the median density value (bs kb^−1^). *P*-value for Mann–Whitney rank test is indicated on the graph. (**e**) Heat map representing the distribution of distances between LFYbs. The LFY-bound regions in *p35S:LFY* were pooled in deciles according to their CFR value (*y* axis shows the groups median CFR value). The *x* axis shows the distance (in bp) separating two 19-bp LFYbs. Only distances <40 bp are shown. (**f**) Boxplot showing the CFR of regions depending on the number of pairs of LFYbs separated by 1 bp (zero, one and two pairs of LFYbs). *P*-values for Mann–Whitney rank test are indicated on the graph.

To test the importance of a functional SAM domain for LFY DNA binding *in vivo*, we performed biological duplicate ChIP sequencing (ChIP-Seq) experiments with *p35S:LFY*, *p35S:LFY*_*TERE*_ and non-transgenic *A. thaliana* (Col-0) 2-week-old seedlings. At this stage, the expression of the endogenous *LFY* is minimal as compared with that of the transgenes, enabling us to use the WT seedlings as a negative control. We chose to use ectopic expression in seedlings so that we could compare LFY and LFY_TERE_ DNA-binding activity in the same tissue, a condition that would not be fulfilled by comparing *lfy* inflorescences expressing LFY or LFY_TERE_. We are aware that, when ectopically expressed, LFY might contact some genomic regions that are not genuine targets. However, it is known that the LFY-bound regions in seedlings and inflorescences significantly overlap and the LFY-binding motifs in the two tissues are extremely similar^17^,^18^. We therefore compared the intensity of LFY binding in *p35S:LFY* and *p35S:LFY*_*TERE*_ for high confidence bound regions identified in *p35S:LFY*. After signal normalization (Supplementary Fig. 6), we found that the TERE mutation drastically reduced the LFY binding *in vivo* (Fig. 5a,b and Supplementary Fig. 7a). For all subsequent analyses, we define the coverage fold reduction (CFR) as the ratio between the LFY and the LFY_TERE_ ChIP-Seq coverages. The CFR values ranged from 1.3 to >150 (Fig. 5a). Ninety-five per cent of the regions have a CFR >3 and 54% have a CFR >10. We also performed the converse analysis by selecting the highest confidence bound regions in *p35S:LFY*_*TERE*_ and comparing their binding with that observed in *p35S:LFY*. Except for a few regions for which the binding by LFY_TERE_ was slightly stronger, most regions were bound better by LFY (Supplementary Fig. 7b). The binding reduction is unlikely to be due to a less efficient recognition of LFY_TERE_ as compared with LFY by the antibody used in the ChIP, as it was raised against LFY-DBD alone and yielded a comparable signal in western blotting on both proteins (Supplementary Fig. 3). The differences in CFR were apparently neither due to alterations to the sequence specificity of AtLFY_TERE_, as the binding site motifs derived from AtLFY and AtLFY_TERE_ ChIP-Seq were very similar (Supplementary Fig. 7c). This result is consistent with previously published SELEX experiments demonstrating that LFY N-terminal domain deletion does not affect LFY DNA-binding specificity^15^,^17^,^24^. Taken together, these findings indicate that LFY SAM domain is crucial for its genome-wide DNA binding *in vivo*.

### LFY-SAM facilitates binding to sites of suboptimal affinity

The general reduction in DNA binding of LFY_TERE_ is probably partially explained by its capacity to bind as a monomer and scatter over the numerous monomeric sites of the genome. However, as illustrated by three examples (Fig. 5b), the reduction in binding by mutants that are unable to oligomerize varies greatly between genomic regions (Fig. 5a). Gene ontology analysis for genes neighbouring the top high- and low-CFR regions did not detect any specific enrichment. We tried to understand whether the nature and number of LFYbs present in a region might help explaining the binding reduction, as our biochemical analysis suggested. To identify LFYbs, we used a previously validated and highly predictive position weight matrix that computes a score between 0 (highest affinity site) and −56 (lowest affinity) for each 19 pb sequence^17^,^26^. To avoid using an arbitrary threshold above which a DNA stretch qualifies as LFYbs, we devised a specific procedure to compute the threshold (see methods). The low threshold we obtained (−25) indicates that LFY-bound regions are enriched in LFYbs of very weak affinity (Supplementary Fig. 8a). When applied to regions bound by LFY_TERE_, the same procedure yielded a higher value (−20) (Supplementary Fig. 8b), suggesting that oligomerization promotes binding to low-affinity sites. Consistent with this hypothesis, the bound regions that lack high-affinity LFYbs tend to have higher CFR than regions with high-affinity LFYbs (Fig. 5c). We also analysed the possible influence of LFYbs density and found that regions with high LFYbs density have higher CFR (Fig. 5d) than regions with less LFYbs. Thus, a functional SAM domain helps LFY to bind to DNA regions with either sub-optimal LFYbs or clusters of LFYbs. To investigate whether these clusters display a specific structure, we characterized the distribution of distances between LFYbs in bound regions. We found that binding sites separated by 1 bp were specifically overrepresented (three- to fourfold) in regions with high CFR (Fig. 5e). Reciprocally, the presence of one or more 1-bp distant sites significantly increased the CFR value (Fig. 5f). These analyses suggest that SAM-mediated oligomerization promotes LFY binding to a specific LFYbs configuration. We confirmed this hypothesis biochemically, by showing in EMSA that a distance of 1 bp between LFYbs most favoured LFY tetrameric binding over other spacing (0, 2, 6 and 11 pb) (Supplementary Fig. 5). Similar results were obtained when we limited the same analyses to the subset of genomic regions that are both bound by LFY in seedlings and by endogenous LFY in inflorescences^18^ (Supplementary Fig. 9). Except for the effect of binding site density that lost its statistical significance, the conclusions are overall very similar despite the smaller size data set. Altogether, these analyses provide evidence that oligomerization through the LFY SAM domain facilitates LFY binding to regions that lack high-affinity binding sites or display multiple and adjacent sites.

### LFY-SAM allows access to closed chromatin regions

**Figure 6:**
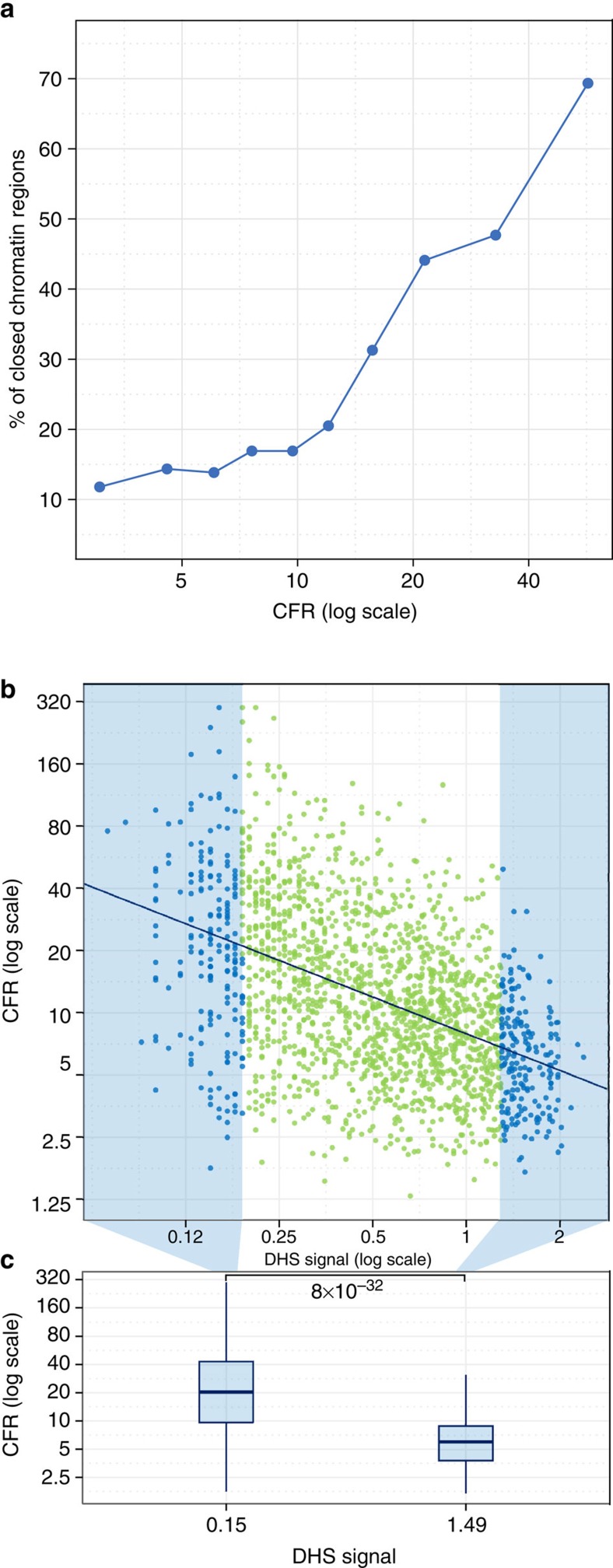
Effect of the chromatin state on the genome-wide LFY DNA binding *in planta*. (**a**) Percentage of closed regions in deciles of p35S:LFY-bound regions sorted according to their CFR. (**b**) Comparison of the CFR of the LFY-bound regions in *p35S:LFY* and the chromatin accessibility in 15-day-old seedlings (DHS signal)^39^. Pearson's correlation test was performed between the CFR and the DHS signal: *r*^2^=21.71%, *P*-value=7.e−106. Data are shown in light blue and green, and fitted values are shown in dark blue. (**c**) Boxplot representing the CFR signal in the regions of highest and lowest DHS (first and last deciles). *P*-value for Mann–Whitney rank test on the two extreme groups is indicated on the graph.

As other SAM domain proteins have been suggested to regulate chromatin status^37^,^38^, we also tested whether the CFR could depend on the accessibility of DNA regions. This genomic feature can be monitored using DNAseI hypersensitivity (DHS) coupled to high-throughput sequencing (DNAseI-Seq). Such data were publicly available for 2-week-old WT *A. thaliana* seedlings^39^, the same developmental stage we used for the ChIP-Seq experiments. We therefore calculated the percentage of opened regions (following the criteria defined in ref. 39) in deciles of LFY-bound regions, sorted according to their CFR. In the low CFR regions, only 10–15% of the LFY-bound regions were closed. Strikingly, this number reached 70% in regions with high CFR (Fig. 6a), suggesting that the chromatin state of the bound regions has a strong impact on the need for LFY oligomerization. To complement this analysis, we studied the correlation between quantitative DHS levels and the CFR. We found that the CFR values remarkably decreased when the accessibility (that is, the DHS level) increased (Fig. 6b) and poorly accessible regions have higher CFR values than accessible ones (Fig. 6c). An independent DNAseI-Seq data set from slightly younger seedlings (7-day-old) yielded similar results (Supplementary Fig. 7d)^40^. Again, these conclusions held true for the subset of genomic regions for which binding by LFY binding has also been validated in inflorescences (Supplementary Fig. 9g–i). Taken together, these results provide strong evidence that LFY binding to closed chromatin regions depends on the presence of a functional SAM domain.

## Discussion

The crystal structure of the conserved LFY N-terminus revealed a SAM oligomerization domain. This finding was unexpected not only because the LFY-N primary sequence has no homology with other SAM domains but also because LFY-N was initially proposed to be a putative leucine zipper dimerization domain^27^. The SAM domain appears to be highly conserved throughout LFY evolution (Fig. 1d and Supplementary Fig. 2d) and probably performs the same oligomerization function in all plant species. Both LFY from *G. biloba* (gymnosperm) and *A. thaliana* (angiosperm) oligomerize. Moreover, chimeric proteins bearing the AtLFY-DBD combined with the SAM from the moss *Physcomitrella patens* or the fern *Ceratopteris richardii* complemented the *A. thaliana lfy* mutant, indicating that the biochemical properties of the SAM domain are evolutionary conserved^41^.

*In planta* experiments revealed that altering the capacity of LFY to oligomerize compromised its floral function and drastically reduced its genome-wide DNA binding. The fact that TF oligomerization could contribute to DNA binding was expected based on the biochemical characterization we performed on LFY, as well as previous work on PRH-Hex^9^ and on TFs from the ETS family^34^. Specifically, our study shows the extent to which genome-wide DNA binding can be altered when oligomerization is disrupted. The single TF for which this question was addressed so far is YAN, a *Drosophila* ETS TF^38^. The analysis of a non-oligomeric version of its SAM domain indicated that YAN oligomerization did not appear to be the primary determinant for its spreading over extended chromatin regions. Another study focused on PRC1, which is not a TF but a SAM domain member of the polycomb repressive complex in mammals. This work showed that mutation of its oligomerization capacity affected its DNA binding in only 12% of its target regions in mouse embryonic cells^37^. Our study thus offers evidence that TF oligomerization can have a profound impact on its genome-wide DNA-binding landscape.

We identified several reasons why the SAM oligomerization domain might influence LFY DNA binding. First, the SAM domain appears to limit LFY binding as a monomer and therefore might prevent LFY scattering over the genome due to nonspecific binding. How the SAM oligomerization domain reduces monomeric binding is unknown. However, it is tempting to speculate that it might impose constraints on the DBD, reducing its ability to interact with sub-optimal sites as a dimer. Second, combining *in vitro* and *in vivo* experiments, we showed that the SAM oligomerization domain facilitates cooperative higher-order LFY complex formation on regions with multiple or low-affinity LFYbs. Even for LFYbs that appear relatively isolated in the genome, it is conceivable that LFY oligomerization could contribute to cooperative binding through DNA looping. Cooperative TF DNA binding was previously shown to be important to trigger developmental switches^42^,^43^ and the development of flowers is one of the best examples of a phase transition in plants. When coupled to clusters of low-affinity binding sites, TF oligomerization was shown to increase the sharpness of expression patterns^44^. Therefore, as levels of LFY progressively increase^45^, oligomerization may induce target genes with specific spatio-temporal patterns depending on their promoter *cis*-element topology (LFYbs affinity and density). For example, the early target *AP1* possesses only a few LFYbs, including one of the highest affinity sites found in the genome (score −7) and might thus be induced early during flower development, whereas genes such as *AP3*, *AG* or *TFL1* that contain a number of lower affinity sites (scores between −10 and −15) are regulated (induced or repressed) later during flower development. SAM-mediated oligomerization could thus contribute to the timing in the expression of LFY target genes. Finally, although we consider it unlikely, we cannot totally exclude that the TERE mutations could have an indirect effect on LFY DNA binding *in vivo*, independent of oligomerization. These mutations could, for example, affect the interactions with yet unidentified regulators that contribute to the efficiency of LFY DNA binding.

By combining ChIP-Seq data with maps of DNA accessibility^39^,^40^, we have also provided evidence for a novel and unexpected role for SAM domain-mediated oligomerization: it enables LFY to access closed chromatin regions. Regulatory regions embedded in closed chromatin are usually poorly accessed by TFs^4^,^46^,^47^. However, a handful of factors, termed ‘pioneer TF', are able to interact with packed chromatin to affect transcriptional activity^48^,^49^. Several of those factors are potent developmental regulators involved in cell programming or reprogramming^49^. LFY is known to play a key role in stem cell emergence and floral fate determination^10^,^15^,^16^. Whether it qualifies as a pioneer TF will require testing its effect on the chromatin status of bound regions^49^. In recent times, the MADS TF SEPALLATA3 and AP1 have also been proposed to play a pioneer role during floral organ development^50^,^51^,^52^. Interestingly, these TFs, as LFY^50^, interact with the several chromatin-remodelling factors^51^ that, once recruited to closed chromatin regions, could contribute to their opening.

Determining the crystal structure of LFY-N not only revealed its identity as a SAM oligomerization domain but also provided the tools to uncover its function during floral development. We provide evidence that oligomerization can have a profound effect on a TF binding landscape by promoting cooperative binding of LFY to DNA, as was proposed for other oligomeric TFs, and, more unexpectedly, also gives LFY access to closed chromatin regions that are notably refractory to TF binding.

## Methods

### Plant growth conditions and transformation

WT, mutants and transgenic lines used are in the *A. thaliana* Columbia-0 background. *A. thaliana* seeds were sown on 0.5 × Murashige and Skoog (Duchefa biochemie) basal salt mixture medium. Plates were stratified 3 days at 4 °C, grown at 22 °C under long-day conditions and seedlings were transferred to soil. *Lfy-12* mutation genotyping was performed as described^35^. *A. tumefaciens* C58C1 pMP90 was used for stable transformation of *lfy-12* heterozygous plants by the floral dip method^53^.

### Plasmid constructions for protein expression in *Escherichia coli*

Plasmids were constructed using primers listed in the Supplementary Table 3. GbLFYΔ (*pETH164*; residues 55–402 from *G. biloba* LFY complementary DNA) was amplified with primers oETH1067 and oETH1068, cloned into the PCR-Blunt vector (Invitrogen) to yield the pCA04 vector and transferred to the *pETM-11* expression vector using NcoI and XhoI restriction sites. GbLFY-SAM (*pETH195*; residues 54–159 from *G. biloba* LFY cDNA) and AtLFY-SAM (*pETH201*; residues 38–151 from *A. thaliana* LFY cDNA) were amplified with the primers oETH1126/oETH1128 and oETH1130/oETH1131, respectively. The PCR products were cloned into the pETM-11 expression vector^54^ using NcoI and XhoI restriction sites, generating fusions with an N-terminal 6 × His tag, cleavable by the tobacco etch virus (TEV) protease. AtLFY (*pETH94*; residues 1–420 from *A. thaliana* LFY cDNA) was amplified with the primer oETH1031/oETH1032, cloned into the PCR-Blunt vector and transferred into the *pET-30a* vector (Novagen) (C-terminal 6 × His tag) using NCoI and XhoI restriction sites.

Mutagenesis was done by site-directed mutagenesis with primers listed in the Supplementary Table 4. GbLFYΔ_TE_ (*pCA21*), GbLFYΔ_RE_ (*pCA22*) and GbLFYΔ_TERE_ (*pCA39*) were derived from *pETH164*; AtLFY_TE_ (*pCA23*), AtLFY_RE_ (*pCA24*) and AtLFY_TERE_ (*pCA25*) were derived from *pETH94*; GbLFY-SAM_TE_ (*pCA15*), GbLFY-SAM_RE_ (*pCA17*) and GbLFY-SAM_TERE_ (*pCA20*) were derived from *pETH195*. All plasmids were verified by appropriate digestions or sequencing.

### Binary vector constructions for *A. thaliana* transformation

*pLFY:LFY*_*TERE*_ (*pCA35*) and *p35S:LFY*_*TERE*_ (*pCA29*) were obtained by inserting a PstI–SalI fragment containing the TERE mutations from *pCA25* in the *pETH29* and *pCA26* (ref. 15), respectively.

### Protein expression and purification

Proteins were expressed using *E. coli* Rosetta2 (DE3) strain (Novagen). Cells were grown in Luria-Bertani medium supplemented with Kanamycin (50 μg ml^−1^) and Chloramphenicol (34 μg ml^−1^) at 37 °C under agitation up to an optical density of 600 nm of 0.6. Betaine (2 mM) was added and cultures were shifted to 17 °C for 1 h before addition of 0.4 mM isopropyl β-D-1-thiogalactopyranoside. After overnight growth at 17 °C, cells were pelleted. Production of selenomethionine (SeMet) GbLFY-SAM for crystallography was carried out in *E. coli* B834 (DE3) (met^−^) strain (Novagen) transformed with the GbLFY-SAM plasmid. The growth of bacteria was initiated in 0.5 l of Luria-Bertani medium supplemented with Kanamycin (50 μg ml^−1^) and Chloramphenicol (34 μg ml^−1^) at 37 °C under agitation up to an optical density of 600 nm of 0.5. Bacteria were harvested by centrifugation and washed with M9 minimal medium. The growth was continued at 37 °C until an optical density of 600 of 0.6 in 1 litre of SeMet buffer (2 × M9 minimal medium+MgSO_4_ (2 mM), FeSO_4_ (25 mg ml^−1^), glucose (4 g l^−1^), vitamins (thiamine, pyridoxine, riboflavin and niacinamide at 1 mg ml^−1^), a mix of all amino acids, except methionine (40 mg ml^−1^), SeMet (40 mg ml^−1^) and antibiotics (34 μg ml^−1^ of Chloramphenicol and 50 μg ml^−1^ of Kanamycin)). The expression of the SeMet protein and growth of the cells at 17 °C were further performed as described above for the GbLFY-SAM protein. All purification steps were performed at 4 °C. All proteins were solubilized and first purified by an affinity chromatography column. The buffer composition used for each protein is given in Supplementary Table 5. Pellets corresponding to 0.5 l culture containing the recombinant protein were sonicated in 50 ml buffer supplemented by one protease inhibitor cocktail tablet Complete EDTA-free (Roche) and centrifuged for 30 min at 20,000 *g*. The clear supernatant was transferred on a column containing 1 ml Ni-Sepharose High Performance resin (GE Healthcare), washed with buffer containing 20 and 40 mM imidazole, and eluted with buffer containing 300 mM imidazole. Eluted fractions were immediately diluted three times in buffer without imidazole and dialysed overnight.

For GbLFY-SAM, GbLFY-SAM_TE_, GbLFY-SAM_RE_, GbLFY-SAM_TERE_ and AtLFY-SAM, after dialysis, the 6 × His tag was cleaved overnight by the TEV protease (5% w/w). The cleavage product was loaded on a Ni-Sepharose High Performance resin to remove the 6 × His tag, the non-cleaved protein and the 6 × His-tagged TEV protease. The protein was eluted with buffer A (Supplementary Table 5) plus 20 mM imidazole. Eluted fractions were applied to a Hi-load Superdex-200 16/60 Prep Grade Column (GE Healthcare) equilibrated with buffer A. After concentration using Amicon Ultra Centrifugal filters (Millipore), the protein concentration was assessed using a NanoDrop-2000 spectrophotometer (Thermo Fisher Scientific Inc.). For crystallography, the SeMet GbLFY-SAM was purified as the native protein.

After dialysis in buffer B, GbLFYΔ, GbLFYΔ_TE_, GbLFYΔ_RE_ and GbLFYΔ_TERE_ proteins were concentrated using Amicon Ultra Centrifugal filters and applied to a Hi-load Superdex-200 16/60 Prep Grade Column equilibrated with buffer C. Protein concentration was assessed using a NanoDrop-2000 spectrophotometer.

After dialysis in buffer D, AtLFY, AtLFY_TE_, AtLFY_RE_ and AtLFY_TERE_ proteins were concentrated using Amicon Ultra Centrifugal filters. Protein concentrations were assessed using the Bradford assay^55^. All proteins were flash frozen in liquid nitrogen and stored at −80 °C.

### Crystal structure determination

Initial crystallization conditions were identified using the high-throughput crystallization platform at EMBL Grenoble (embl.fr/htxlab). The optimum condition was obtained at 20 °C with the hanging drop vapour diffusion method by mixing 1 μl GbLFY-N, native or Se-Met substituted, at 2.5 mg ml^−1^ with 1 μl of reservoir solution containing 25 mM Tris-HCl pH 8.8 and 40 mM ammonium sulfate. Crystals were cryoprotected by plunging into liquid nitrogen, after incubation in well conditions to which 20% glycerol had been supplemented. The structure was solved by the single-wavelength anomalous dispersion method from Se-Met substituted protein). A highly redundant data set was collected on the microfocus beamline ID23-2 (ref. 56). The selenium substructure was determined by SHELXC/D^57^ and the selenium positions were refined in SHARP^58^. Phases were calculated, solvent-flattened maps were obtained with PIRATE^59^ and a partial model was automatically built with BUCCANEER^60^. The model was manually extended into the experimental electron density map and the resultant model was then used as a search model for molecular replacement into the higher-resolution native data (preserving and extending the reflections previously selected for the free set). Successive rounds of model improvement in COOT^61^ and refinement in BUSTER^62^ were then performed. The built region in the two monomers present in the asymmetric unit comprised residues 54–134 and 56–133, respectively.

### Western blotting

*A. thaliana* leaves from 15-day-old seedlings grown under long-day conditions were harvested and one leaf from six to eight seedlings were pooled for each transgenic line. The leaves were flash frozen in liquid nitrogen, mechanically crushed, immediately suspended in 80 μl denaturing buffer (50 mM Tris-HCl pH 6.8, 10% w/v glycerol, 1% w/v SDS, 0.0025% w/v Bromophenol blue and 0.4% w/v dithiothreitol) for 10 mg of fresh matter and denatured for 5 min at 95 °C. The samples were run on a SDS–PAGE gel and transferred on a nitrocellulose membrane (Immobilon P transfer membrane, Millipore) for blotting. The equivalent of 18.75 and 12.5 mg of fresh matter were used for anti-LFY and anti-KARI blots, respectively. Five nanograms of recombinant LFY protein (LFYΔ, produced in *E. coli*) were used as a positive control. Two anti-LFY antibodies were used: JA70 raised in rabbit against the recombinant LFY protein^17^ (used at 1/6,000 dilution in Supplementary Fig. 3a) and 4028 (ref. 17) raised in rabbit against the LFY C-terminal amino acids 223–424 (BioGenes). Anti-KARI antibodies (used at 1/3,000) were described in ref. 63. Horseradish peroxidase-conjugated AffiniPure goat anti-rabbit IgG (used at 1/15,000, 111-035-144, Jackson ImmunoResearch) was used as secondary antibody for detection. For Supplementary Fig. 3b, antibodies J70 and 4028 were used at 1/25,000 followed by the secondary antibody at 1/50,000 dilution. Revelation was done using the Pierce ECL2 Western Blotting Substrate kit (Thermo Scientific) and scanned on a Typhoon 9400 scanner (Molecular Dynamics). Uncropped western blottings and protein ladders are presented as Supplementary Fig. 10.

### Protein sequence and structure analysis

Three-dimensional structure images were done using PyMOL (www.pymol.org); sequence alignment was done using Multalin (http://multalin.toulouse.inra.fr/multalin/) and visualized using ESPrit (http://espript.ibcp.fr/ESPript/ESPript/). Root mean squared deviations were computed using the Dali server (http://ekhidna.biocenter.helsinki.fi/dali_server/). Surface conservation was computed using the Consurf server (http://consurf.tau.ac.il/; (ref. 64)).

### Electrophoretic mobility shift assay

Oligonucleotides used for EMSA are listed in Supplementary Table 6. For *AP1*, *AP1m1*, *AP1m2*, *AGI-II* and *S-AGI*, complementary single-stranded oligonucleotides were annealed in annealing buffer (10 mM Tris pH 7.5, 150 mM NaCl and 1 mM EDTA). The resulting double-stranded DNA with a protruding G was fluorescently labelled by end filling: 4 pmol of double-stranded DNA was incubated with 1 unit of Klenow fragment polymerase (Ozyme) and 8 pmol Cy3-dCTP or Cy5-dCTP (GE Healthcare) in Klenow buffer during 2 h at 37 °C, followed by 10 min enzyme inactivation at 65 °C. Binding reactions were performed in 20 μl binding buffer (20 mM Tris-HCl pH 7.5, 150 mM NaCl, 1% glycerol, 0.25 mM EDTA, 2 mM MgCl_2_, 0,01% Tween-20 and 3 mM TCEP) with 10 nM labelled probe, 1 × (28 ng ml^−1^) fish sperm DNA (Roche) as nonspecific competitor and 25–500 nM proteins.

Competition assays were performed in duplicates and 1–100 × fish sperm DNA (Roche) was used in the binding reaction. Signal quantification was performed in using ImageLab v2.0.1 (Bio-Rad Laboratories). Signal of each protein–DNA complex was quantified relatively to total DNA signal. For Fig. 4e, each binding reaction was performed in triplicate. Uncropped gels are presented as Supplementary Fig. 10.

### SEC and SEC-MALLS

The molecular mass of GbLFY-SAM and AtLFY-SAM was estimated at 4 °C using a Superdex-200 10/300GL column (GE Healthcare), equilibrated with buffer A and calibrated with low- and high-molecular-weight protein standards (gel filtration calibration kit; GE Healthcare). Accurate molecular mass determination using SEC-MALLS was carried out with a Superdex-200 10/300GL column (GE Healthcare). GbLFY-SAM, WT and mutants were analysed in buffer A. Protein–DNA complexes containing GbLFYΔ, WT or mutants and *AP1* DNA were analysed in 20 mM Tris-HCl pH 8, 150 mM NaCl, 0.25 mM EDTA, 2 mM MgCl_2_ and 1 mM TCEP. Separations were performed at 20 °C with a flow rate of 0.5 ml min^−1^. Elutions were monitored by using a DAWN-EOS detector with a laser emitting at 690 nm for online MALLS measurement (Wyatt Technology Corp., Santa Barbara, CA) and with a RI2000 detector for online refractive index measurements (Schambeck SFD). Molecular mass calculations were performed using the ASTRA software using a refractive index increment (d*n*/d*c*) of 0.185 ml g^−1^.

### Chromatin immunoprecipitation sequencing

Fifteen-day-old seedling from lines CA26 #15 (*p35S:LFY*), CA29 #17 (*p35S:LFY*_*TERE*_) and Col-0 were grown on 0.5 × Murashige and Skoog medium under long-day conditions. The experiment was performed twice to produce biological replicates. Seedlings (∼1 g) were fixed with 1% formaldehyde in MC buffer (10 mM sodium phosphate pH 7.0, 50 mM NaCl and 0.1 M sucrose) for 1 h under vacuum. Fixation was stopped with 0.125 M glycine, followed by three washes with MC buffer. The tissue was ground in liquid nitrogen, the powder was suspended in 15 ml M1 buffer (10 mM sodium phosphate pH 7.0, 0.1 M NaCl, 1 M 2-methyl 2,4-pentanediol, 10 mM β-mercaptoethanol, Complete Protease Inhibitor Cocktail (Roche Diagnostics GmbH, Mannhein, Germany)). The slurry was filtrated three times through 55-μm mesh (Miracloth, Calbiochem) and centrifuged at 1,000 *g* for 10 min at 4 °C. Filtration and centrifugation were repeated twice. Subsequent steps were at 4 °C, unless indicated otherwise. The pellet was washed five times with 1 ml M2 buffer (M1 buffer with 10 mM MgCl_2_ and 0.5% Triton X-100), once with 1 ml M3 buffer (M1 without 2-methyl 2,4-pentanediol) and resuspended in 1 ml Sonic buffer (10 mM sodium phosphate pH 7.0, 0.1 M NaCl, 0.5% Sarkosyl, 10 mM EDTA, Complete Protease Inhibitor Cocktail and 1 mM PEFA BLOC SC (Roche Diagnostics)), and sonicated with a Focused-ultrasonicator S2 (Covaris) (Duty cycle: 20%, intensity: 5, cycles per burst: 200, cycle time: 2 min). After sonication, the suspension was centrifuged (microcentrifuge, top speed) for 5 min and the supernatant was mixed with one volume of IP buffer (50 mM Hepes pH 7.5, 150 mM KCl, 5 mM MgCl_2_, 10 μM ZnSO_4_, 1% Triton X-100 and 0.05% SDS). The solubilized chromatin was incubated overnight with 2.5 μl anti-LFY serum antibody 4028. The immunoprotein–chromatin complexes were captured by incubation with protein A-agarose beads (Santa Cruz Biotechnology) for 1 h on a rotating wheel. The protein A-agarose beads were washed five times with 1 ml IP buffer for 10 min at room temperature. Beads were vortexed for 30 s with 100 μl cold glycine elution buffer (0.1 M glycine, 0.5 M NaCl and 0.05% Tween-20 pH 2.8) and pelleted in a microfuge (room temperature, 1 min, top speed). The supernatant was mixed with 50 μl of 1 M Tris pH 9, to neutralize the eluant. Elution and neutralization were repeated twice. The eluted sample was spun at top speed for 2 min in a microcentrifuge at room temperature. The eluate was treated with 1 μl RNase A/T1 mix 10 mg ml^−1^ (Fermentas) and 1.5 μl Proteinase K, recombinant, PCR grade (Roche). After overnight incubation, a second aliquot of Proteinase K was added and incubated at 65 °C for 6 h. DNA was purified using Minelute columns (Qiagen). Libraries for high-throughput sequencing were prepared using standard Illumina protocols. Deep sequencing was performed on an Illumina GAIIx instrument following the manufacturer's instructions.

### Bioinformatics analyses

Bioinformatics analyses were performed using the SHORE suite (version 0.9.3) (ref. 65) and scripts in python (version 2.7.3) using BioPython (version 1.60), pybedtools (version 0.6.4) and Matplotlib (version 1.2.0) modules. All statistical analyses were managed using R (version 3.0.2) and figures were generated using the ggplot2 module (version 1.0.0). All scripts are available on request.

Read mapping and duplicates removal were performed using the SHORE pipeline (preprocess, import, mapflowcell subprocesses) using -H 1,1 -M 0,4 -X 130 -B 1 as parameters. The number of unique reads mapped is indicated in the Supplementary Table 7. Peakfinding was performed with the shore peak subprogramme using –B 1 as parameter. We selected as bound regions all peaks with a BH-FDR-*q*<10^−7^ in all four comparisons of the two replicates against the two controls. A python script was used to build bound peak bed files from SUMMARY.txt shore peak output. We obtained 1,954 peaks for *p35S:LFY* and 176 for *p35S:LFY*_*TERE*_. Corresponding bed files with peak rank and CFR values have been uploaded on the GEO database.

For each genomic position, the read number corresponds to the number of reads extended by 130 bp covering this position. For each bound region (ChIP-Seq peak), the coverage is defined as the area above the read number curve.

The normalization procedure was performed in two steps based on peak coverage comparisons. In *p35S:LFY* and *p35S:LFY*_*TERE*_ we used peaks detected by SHORE, and in the Col control we used a python script as rudimentary peak finder using 20 as a cutoff value for signal (Supplementary Fig. 6). First, we performed intra genotype normalization between ChIP-Seq replicates from the same genotype (Col, *p35S:LFY* or *p35S:LFY*_*TERE*_). For each genotype, we used the most significant peaks and plotted the coverage values of one replicate against the other. The regression coefficient *m* as shown in Supplementary Fig. 6 was used to normalize one replicate against the other.

Replicates were then fused by calculating a normalized read count at each position


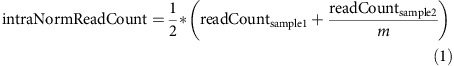


Second, inter genotype normalization was performed based on the background peaks detected in the Col control sample. For each background peak, coverages were compared between normalized *p35S:LFY* or *p35S:LFY*_*TERE*_ and the Col control sample (Supplementary Fig. 6b), and the regression coefficient *m'* was computed and used for final normalization:


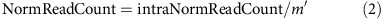


for p35S:LFY and p35S:LFY_TERE_ ChIP-Seq. All coefficients used are listed in Supplementary Table 8.

To calculate read coverages (based on ChIP-Seq or DnaseI-Seq signals), we selected the genomic information included in ‘.wig' or ‘.bedgraph files' at bound regions using the bedtools intersect subprogram (-wa –wb option) followed by a python script that calculates the coverage of each of those genomic regions. The CFR due to the TERE mutation was computed with R using equation (3) on normalized wig files for each bound regions.


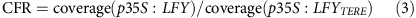


When computing CFR for the 1,954 LFY-bound regions (Fig. 5a), the very low coverage in *p35S:LFY*_*TERE*_ for two regions was leading to very high or infinite CFR values. Those two CFR values were arbitrarily set up to 300.

The chromatin information about chromatin state (open versus closed) for each bound region was retrieved with a python script from *dhleaf.txt* and *dhflower.txt* files from ref. 39. A bound region was considered open when its centred half was entirely open.

To find the LFYBs in ChIP-Seq peaks, we used a previously optimized position weight matrix^17^ and python script computing a score between −56 and 0 to each 19-pb sequence. Zero corresponds to the site of highest affinity and −56 corresponds to the lowest affinity. To determine what score threshold should be used for binding-site identification, we compared bound regions with a negative set of unbound regions. The negative set was built with a python script that takes a bed file with bound regions as input and randomly selects in the *Arabidopsis* genome regions of same size, similar GC content and with various annotation type (CDS, intron, upstream, downstream or intergenic). The LFYbs scores for each bound and unbound regions were calculated. We then used an R script to compute the density of LFYbs in sliding score windows (going from [0; −1] to [−55; −56]). As shown in Supplementary Fig. 8a, the density of LFYbs is significantly higher in the positive set as compared with the negative one for scores better than −25, as judged using a Mann–Whitney rank test on each window of scores (*P*-value<0.05). The corresponding binding site density might appear surprisingly high but we believe that our score threshold calculation is more valid than commonly used arbitrary cutoff score values. Moreover, our analyses were robust to threshold change within a reasonable range [−20, −26].

For the heat map of interdistances (Fig. 5e), we used a python script to divide regions in deciles according to their CFR, calculate all distances between LFYbs and plot their number within the decile. To perform the analysis presented in Supplementary Fig. 9, we kept only the 436 regions found as bound in 35S:LFY seedlings that intersected with regions bound by LFY in inflorescences according to ref. 18 and performed the whole pipeline of analysis. R was used for all figures and statistical analyses, except that Supplementary Fig. 6 was generated by the Matplotlib module of python.

## Additional information

**How to cite this article:** Sayou, C. *et al*. A SAM oligomerization domain shapes the genomic binding landscape of the LEAFY transcription factor. *Nat. Commun.* 7:11222 doi: 10.1038/ncomms11222 (2016).

## Supplementary Material

Supplementary Information Supplementary Figures 1-10, Supplementary Tables 1-7, and Supplementary References

## Figures and Tables

**Table 1:** Crystallographic data collection and refinement statistics.

	**GbLFY-N (Native)**	**GbLFY-N (Se-Met)**
*Data collection*		
Wavelength (Å)	0.872	0.872
Resolution (Å) range	52.34–2.25 (2.33–2.25)	100–2.61 (2.77–2.61)
Space group	*P* 6_5_	*P* 6_5_
Cell dimensions (Å,°)	*a*=*b*=81.1, *c*=78.5	*a*=*b*=80.6, *c*=78.5
Number of total reflections	57,705	83,685
Number of unique reflections	14,004	8,879
Multiplicity	4.1(3.9)	9.4 (9.2)
Completeness (%)	99.3 (99.0)	99.6 (97.5)
Mean I/*σ*(I)	15.67 (2.89)	15.28 (2.54)
*R*-sym	0.069 (0.514)	0.127 (0.915)
		
*Refinement statistics*		
Wilson B-factor	44.1	35.85
*R*-factor	0.1859 (0.2564)	
*R*-free	0.2298 (0.2767)	
Number of atoms	1,462	
Macromolecule	1,335	
Ligands	25	
Water	102	
Protein residues	163	
Root mean square (bonds)	0.016	
Root mean square (angles)	1.78	
Ramachandran favoured (%)	99	
Ramachandran outliers (%)	0	
Clashscore	13.40	
Average B-factor	46.80	
Macromolecule	46.00	
Solvent	51.80	

Statistics for the highest-resolution shell are shown in parentheses.

## References

[b1] Spitz F. & Furlong E. E. M. Transcription factors: from enhancer binding to developmental control. Nat. Rev. Genet. 13, 613–626 (2012) . 22868264 10.1038/nrg3207

[b2] Shlyueva D., Stampfel G. & Stark A. Transcriptional enhancers: from properties to genome-wide predictions. Nat. Rev. Genet. 15, 272–286 (2014) . 24614317 10.1038/nrg3682

[b3] Weirauch M. T. & Hughes T. R. in A Handbook of Transcription Factors 52, 25–73 (2011) . 10.1007/978-90-481-9069-0_3 21557078

[b4] Slattery M. . Absence of a simple code: how transcription factors read the genome. Trends Biochem. Sci. 39, 381–399 (2014) . 25129887 10.1016/j.tibs.2014.07.002 PMC4149858

[b5] Bienz M. Signalosome assembly by domains undergoing dynamic head-to-tail polymerization. Trends Biochem. Sci. 39, 487–495 (2014) . 25239056 10.1016/j.tibs.2014.08.006

[b6] Hollenhorst P. C., McIntosh L. P. & Graves B. J. Genomic and biochemical insights into the specificity of ETS transcription factors. Annu. Rev. Biochem. 80, 437–471 (2011) . 21548782 10.1146/annurev.biochem.79.081507.103945 PMC5568663

[b7] Nanao M. H. . Structural basis for oligomerization of auxin transcriptional regulators. Nat. Commun. 5, 3617 (2014) . 24710426 10.1038/ncomms4617

[b8] Katsani K. R., Hajibagheri M. A. N. & Verrijzer C. P. Co-operative DNA binding by GAGA transcription factor requires the conserved BTB / POZ domain and reorganizes promoter topology. EMBO J. 18, 698–708 (1999) . 9927429 10.1093/emboj/18.3.698 PMC1171162

[b9] Williams H., Jayaraman P.-S. & Gaston K. DNA wrapping and distortion by an oligomeric homeodomain protein. J. Mol. Biol. 383, 10–23 (2008) . 18755198 10.1016/j.jmb.2008.08.004

[b10] Moyroud E., Tichtinsky G. & Parcy F. The LEAFY floral regulators in angiosperms: conserved proteins with diverse roles. J. Plant Biol. 52, 177–185 (2009) .

[b11] Moyroud E., Kusters E., Monniaux M., Koes R. & Parcy F. LEAFY blossoms. Trends Plant Sci. 15, 346–352 (2010) . 20413341 10.1016/j.tplants.2010.03.007

[b12] Irish V. F. The flowering of *Arabidopsis* flower development. Plant J. 61, 1014–1028 (2010) . 20409275 10.1111/j.1365-313X.2009.04065.x

[b13] Chandler J. W. Floral meristem initiation and emergence in plants. Cell. Mol. Life Sci. 69, 3807–3818 (2012) . 22573183 10.1007/s00018-012-0999-0 PMC11115123

[b14] Posé D., Yant L. & Schmid M. The end of innocence: flowering networks explode in complexity. Curr. Opin. Plant Biol. 15, 45–50 (2012) . 21974961 10.1016/j.pbi.2011.09.002

[b15] Chahtane H. . A variant of LEAFY reveals its capacity to stimulate meristem development by inducing RAX1. Plant J. 74, 678–689 (2013) . 23445516 10.1111/tpj.12156

[b16] Yamaguchi N. . A molecular framework for auxin-mediated initiation of flower primordia. Dev. Cell 24, 271–282 (2013) . 23375585 10.1016/j.devcel.2012.12.017

[b17] Moyroud E. . Prediction of regulatory interactions from genome sequences using a biophysical model for the *Arabidopsis* LEAFY transcription factor. Plant Cell 23, 1293–1306 (2011) . 21515819 10.1105/tpc.111.083329 PMC3101549

[b18] Winter C. M. . LEAFY target genes reveal floral regulatory logic, cis motifs, and a link to biotic stimulus response. Dev. Cell 20, 430–443 (2011) . 21497757 10.1016/j.devcel.2011.03.019

[b19] Weigel D. & Nilsson O. A developmental switch sufficient for flower initiation in diverse plants. Nature 377, 495–500 (1995) . 7566146 10.1038/377495a0

[b20] Gallois J.-L., Nora F. R., Mizukami Y. & Sablowski R. WUSCHEL induces shoot stem cell activity and developmental plasticity in the root meristem. Genes Dev. 18, 375–380 (2004) . 15004006 10.1101/gad.291204 PMC359391

[b21] Risseeuw E. . An activated form of UFO alters leaf development and produces ectopic floral and inflorescence meristems. PLoS ONE 8, e83807 (2013) . 24376756 10.1371/journal.pone.0083807 PMC3871548

[b22] Kaufmann K., Pajoro A. & Angenent G. C. Regulation of transcription in plants: mechanisms controlling developmental switches. Nat. Rev. Genet. 11, 830–842 (2010) . 21063441 10.1038/nrg2885

[b23] Engelhorn J., Blanvillain R. & Carles C. C. Gene activation and cell fate control in plants: a chromatin perspective. Cell. Mol. Life Sci. 71, 3119–3137 (2014) . 24714879 10.1007/s00018-014-1609-0 PMC11113918

[b24] Sayou C. . A promiscuous intermediate underlies the evolution of LEAFY DNA binding specificity. Science 343, 645–648 (2014) . 24436181 10.1126/science.1248229

[b25] Hamès C. . Structural basis for LEAFY floral switch function and similarity with helix-turn-helix proteins. EMBO J. 27, 2628–2637 (2008) . 18784751 10.1038/emboj.2008.184 PMC2567413

[b26] Minguet E. G., Segard S., Charavay C. & Parcy F. MORPHEUS, a webtool for transcription factor binding analysis using position weight matrices with dependency. PLoS ONE 10, e0135586 (2015) . 26285209 10.1371/journal.pone.0135586 PMC4540572

[b27] Siriwardana N. S. & Lamb R. S. A conserved domain in the N-terminus is important for LEAFY dimerization and function in *Arabidopsis thaliana*. Plant J. 71, 736–749 (2012) . 22507399 10.1111/j.1365-313X.2012.05026.x

[b28] Qiao F. & Bowie J. U. The many faces of SAM. Sci. STKE 2005, re7 (2005) . 15928333 10.1126/stke.2862005re7

[b29] Meruelo A. D. & Bowie J. U. Identifying polymer-forming SAM domains. Proteins 74, 1–5 (2009) . 18831011 10.1002/prot.22232 PMC2605191

[b30] Kim C. a . Polymerization of the SAM domain of TEL in leukemogenesis and transcriptional repression. EMBO J. 20, 4173–4182 (2001) . 11483520 10.1093/emboj/20.15.4173 PMC149168

[b31] Ikeda-Kawakatsu K., Maekawa M., Izawa T., Itoh J.-I. & Nagato Y. ABERRANT PANICLE ORGANIZATION 2/RFL, the rice ortholog of *Arabidopsis* LEAFY, suppresses the transition from inflorescence meristem to floral meristem through interaction with APO1. Plant J. 69, 168–180 (2012) . 21910771 10.1111/j.1365-313X.2011.04781.x

[b32] Levin J. Z. & Meyerowitz E. M. UFO: an *Arabidopsis* gene involved in both floral meristem and floral organ development. Plant Cell 7, 529–548 (1995) . 7780306 10.1105/tpc.7.5.529 PMC160802

[b33] Weigel D., Alvarez J., Smyth D. R., Yanofsky M. F. & Meyerowitz E. M. LEAFY controls floral meristem identity in *Arabidopsis*. Cell 69, 843–859 (1992) . 1350515 10.1016/0092-8674(92)90295-n

[b34] Green S. M., Coyne H. J., McIntosh L. P. & Graves B. J. DNA binding by the ETS protein TEL (ETV6) is regulated by autoinhibition and self-association. J. Biol. Chem. 285, 18496–18504 (2010) . 20400516 10.1074/jbc.M109.096958 PMC2881775

[b35] Benlloch R. . Integrating long-day flowering signals: a LEAFY binding site is essential for proper photoperiodic activation of APETALA1. Plant J. 67, 1094–1102 (2011) . 21623976 10.1111/j.1365-313X.2011.04660.x

[b36] Zhang J. . Sterile alpha motif domain-mediated self-association plays an essential role in modulating the activity of the *Drosophila* ETS family transcriptional repressor Yan. Mol. Cell. Biol. 30, 1158–1170 (2010) . 20048052 10.1128/MCB.01225-09 PMC2820895

[b37] Isono K. . SAM domain polymerization links subnuclear clustering of PRC1 to gene silencing. Dev. Cell 26, 565–577 (2013) . 24091011 10.1016/j.devcel.2013.08.016

[b38] Webber J. L. . The relationship between long-range chromatin occupancy and polymerization of the *Drosophila* ETS family transcriptional repressor Yan. Genetics 193, 633–649 (2013) . 23172856 10.1534/genetics.112.146647 PMC3567750

[b39] Zhang W., Zhang T., Wu Y. & Jiang J. Genome-wide identification of regulatory DNA elements and protein-binding footprints using signatures of open chromatin in *Arabidopsis*. Plant Cell 24, 2719–2731 (2012) . 22773751 10.1105/tpc.112.098061 PMC3426110

[b40] Sullivan A. M. . Mapping and dynamics of regulatory DNA and transcription factor networks in *A. thaliana*. Cell Rep. 8, 2015–2030 (2014) . 25220462 10.1016/j.celrep.2014.08.019

[b41] Maizel A. . The floral regulator LEAFY evolves by substitutions in the DNA binding domain. Science 308, 260–263 (2005) . 15821093 10.1126/science.1108229

[b42] Cherry J. L. & Adler F. R. How to make a biological switch. J. Theor. Biol. 203, 117–133 (2000) . 10704297 10.1006/jtbi.2000.1068

[b43] Lebrecht D. . Bicoid cooperative DNA binding is critical for embryonic patterning in *Drosophila*. Proc. Natl Acad. Sci. USA 102, 13176–13181 (2005) . 16150708 10.1073/pnas.0506462102 PMC1201621

[b44] Segal E., Raveh-Sadka T., Schroeder M., Unnerstall U. & Gaul U. Predicting expression patterns from regulatory sequence in *Drosophila* segmentation. Nature 451, 535–540 (2008) . 18172436 10.1038/nature06496

[b45] Blázquez M. A., Soowal L. N., Lee I. & Weigel D. LEAFY expression and flower initiation in *Arabidopsis*. Development 124, 3835–3844 (1997) . 9367439 10.1242/dev.124.19.3835

[b46] Sherwood R. I. . Discovery of directional and nondirectional pioneer transcription factors by modeling DNase profile magnitude and shape. Nat. Biotechnol. 32, 171–178 (2014) . 24441470 10.1038/nbt.2798 PMC3951735

[b47] Voss T. C. & Hager G. L. Dynamic regulation of transcriptional states by chromatin and transcription factors. Nat. Rev. Genet. 15, 69–81 (2014) . 24342920 10.1038/nrg3623 PMC6322398

[b48] Guertin M. J. & Lis J. T. Mechanisms by which transcription factors gain access to target sequence elements in chromatin. Curr. Opin. Genet. Dev. 23, 116–123 (2013) . 23266217 10.1016/j.gde.2012.11.008 PMC3651763

[b49] Iwafuchi-doi M. & Zaret K. S. Pioneer transcription factors in cell reprogramming. 28, 2679–2692 (2014) . 10.1101/gad.253443.114 PMC4265672 25512556

[b50] Wu M.-F. . SWI2/SNF2 chromatin remodeling ATPases overcome polycomb repression and control floral organ identity with the LEAFY and SEPALLATA3 transcription factors. Proc. Natl Acad. Sci. USA 109, 3576–3581 (2012) . 22323601 10.1073/pnas.1113409109 PMC3295252

[b51] Smaczniak C. . Characterization of MADS-domain transcription factor complexes in *Arabidopsis* flower development. Proc. Natl Acad. Sci. 109, 1560–1565 (2012) . 22238427 10.1073/pnas.1112871109 PMC3277181

[b52] Pajoro A. . Dynamics of chromatin accessibility and gene regulation by MADS-domain transcription factors in flower development. Genome Biol. 15, R41 (2014) . 24581456 10.1186/gb-2014-15-3-r41 PMC4054849

[b53] Zhang X., Henriques R., Lin S.-S., Niu Q.-W. & Chua N.-H. Agrobacterium-mediated transformation of *Arabidopsis thaliana* using the floral dip method. Nat. Protoc. 1, 641–646 (2006) . 17406292 10.1038/nprot.2006.97

[b54] Dümmler A., Lawrence A.-M. & de Marco A. Simplified screening for the detection of soluble fusion constructs expressed in E. coli using a modular set of vectors. Microb. Cell Fact. 4, 34 (2005) . 16351710 10.1186/1475-2859-4-34 PMC1326211

[b55] Bradford M. M. A rapid and sensitive method for the quantitation of microgram quantities of protein utilizing the principle of protein-dye binding. Anal. Biochem. 72, 248–254 (1976) . 942051 10.1016/0003-2697(76)90527-3

[b56] Flot D. . The ID23-2 structural biology microfocus beamline at the ESRF. J. Synchrotron Radiat. 17, 107–118 (2010) . 20029119 10.1107/S0909049509041168 PMC3025444

[b57] Sheldrick G. M. Experimental phasing with SHELXC/D/E: combining chain tracing with density modification. Acta Crystallogr. D Biol. Crystallogr. 66, 479–485 (2010) . 20383001 10.1107/S0907444909038360 PMC2852312

[b58] Bricogne G., Vonrhein C., Flensburg C., Schiltz M. & Paciorek W. Generation, representation and flow of phase information in structure determination: recent developments in and around SHARP 2.0. Acta Crystallogr. D Biol. Crystallogr. 59, 2023–2030 (2003) . 14573958 10.1107/s0907444903017694

[b59] Cowtan K. Generic representation and evaluation of properties as a function of position in reciprocal space. J. Appl. Crystallogr. 35, 655–663 (2002) .

[b60] Cowtan K. The Buccaneer software for automated model building. 1. Tracing protein chains. Acta Crystallogr. D Biol. Crystallogr. 62, 1002–1011 (2006) . 16929101 10.1107/S0907444906022116

[b61] Emsley P. & Cowtan K. Coot: model-building tools for molecular graphics. Acta Crystallogr. D Biol. Crystallogr. 60, 2126–2132 (2004) . 15572765 10.1107/S0907444904019158

[b62] Bricogne G. . BUSTER. Available at http://www.globalphasing.com (2011) .

[b63] Dumas R., Joyard J. & Douce R. Purification and characterization of acetohydroxyacid reductoisomerase from spinach chloroplasts. Biochem. J. 262, 971–976 (1989) . 2590180 10.1042/bj2620971 PMC1133368

[b64] Ashkenazy H., Erez E., Martz E., Pupko T. & Ben-Tal N. ConSurf 2010: calculating evolutionary conservation in sequence and structure of proteins and nucleic acids. Nucleic Acids Res. 38, W529–W533 (2010) . 20478830 10.1093/nar/gkq399 PMC2896094

[b65] Ossowski S. . Sequencing of natural strains of *Arabidopsis thaliana* with short reads. Genome Res. 18, 2024–2033 (2008) . 18818371 10.1101/gr.080200.108 PMC2593571

[b66] Sathyamurthy A., Freund S. M. V, Johnson C. M., Allen M. D. & Bycroft M. Structural basis of p63α SAM domain mutants involved in AEC syndrome. FEBS J. 278, 2680–2688 (2011) . 21615690 10.1111/j.1742-4658.2011.08194.x

