# Supplementary material for: A SAM oligomerization domain shapes the genomic binding landscape of the LEAFY transcription factor
Source: Nat Commun. 2016 Apr 21;7:11222. doi: 10.1038/ncomms11222 (PMC4844672; doi:10.1038/ncomms11222)
Supplement: Supplementary Information — Supplementary Figures 1-10, Supplementary Tables 1-7, and Supplementary References [file ncomms11222-s1.pdf]

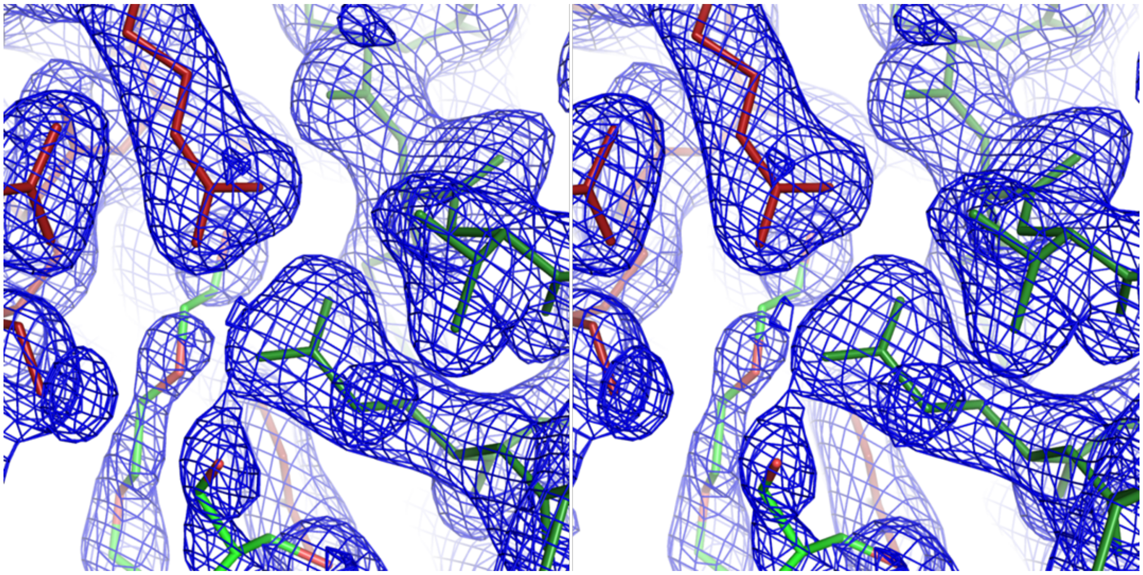

Supplementary Figure 1: Stereo image of a portion of the electron density map of the SAM domain.

Stereoscopic view of the model phased 2Fo-Fc electron density map superimposed over the final refined model.

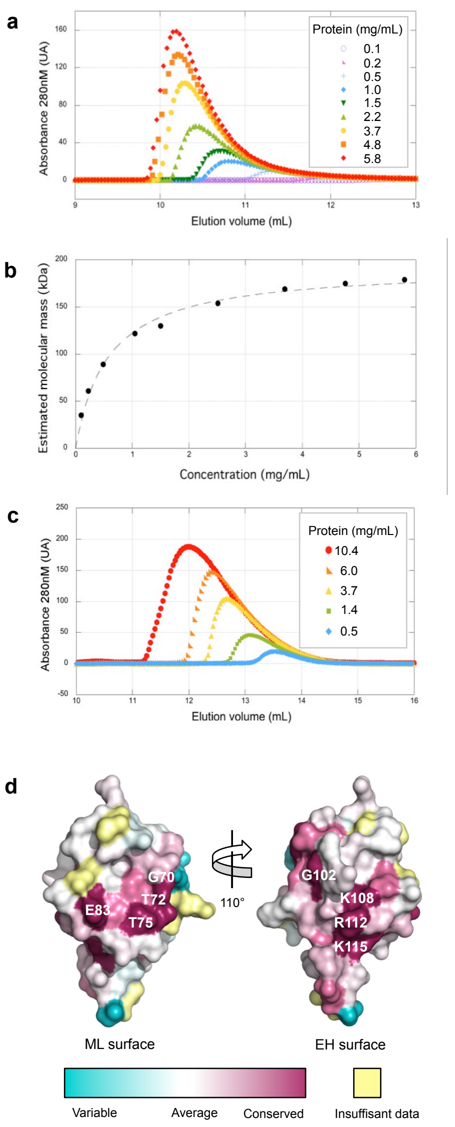

Supplementary Figure 2: Oligomeric states of GbLFY-SAM and AtLFY-SAM and amino acid conservation of the SAM domain.

(a) SEC analysis of GbLFY-SAM at different concentrations.

(b) Apparent molecular weights (MWs) of GbLFY-SAM at different concentrations. MWs were calculated using elution volumes from (a) and a calibration curve based on a set of protein standards. This plot shows that at high protein concentration, the size of the oligomer in solution reaches a plateau value, which, as determined by SEC-MALLS corresponds to an octamer (Supplementary Table 1).

(c) SEC analysis of AtLFY-SAM at different concentrations.

(d) Amino acid conservation of LFY-SAM mapped on the surface of GbLFY-SAM structure. LFY sequences from 18 species from algae to angiosperms (*A. thaliana*, *O. sativa*, *T. aestivum*, *Z. mays*, *P. hybrida*, *A. trichopoda*, *G. biloba*, *P. radiata*, *W. mirabilis*, *M. domestica*, *A. lygodiifolia*, *B. multifidum*, *C. richardii*, *P. patens*, *N. aenigmaticus*, *Cylindrocystis sp*, *C. scutata*, *K. subtile*) were aligned and the surface conservation was computed using the ConSurf server (http://consurf.tau.ac.il/; [1]).

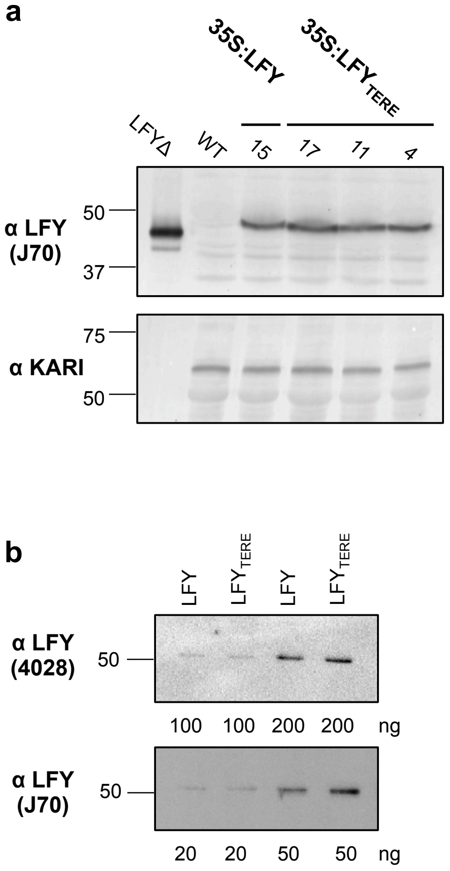

Supplementary Figure 3: LFY and LFY$_{TERE}$ proteins are expressed at similar level in *p35S:LFY* and *p35S:LFY$_{TERE}$* plants used for ChIP-Seq experiments and are detected similarly by LFY antibodies.

A) Western blot on *A. thaliana* leaves extracts from 15 day-old seedlings grown under long day conditions. Single leaves from 6 to 8 seedlings were pooled for each transgenic line (transgenic line identifiers are given above each lane). Anti-KARI antibody was used as control [2]. The molecular weight deduced from standard protein migration is indicated on the left. 5 ng of recombinant LFY protein (LFYΔ, produced in *E. coli*) was used as a positive control.

B) Western blot on LFY and LFY$_{TERE}$ recombinant proteins with the antibodies (α LFY) used in the ChIP-Seq (4028) or on panel a (J70). Each of these antibodies recognized similarly LFY and LFY$_{TERE}$.

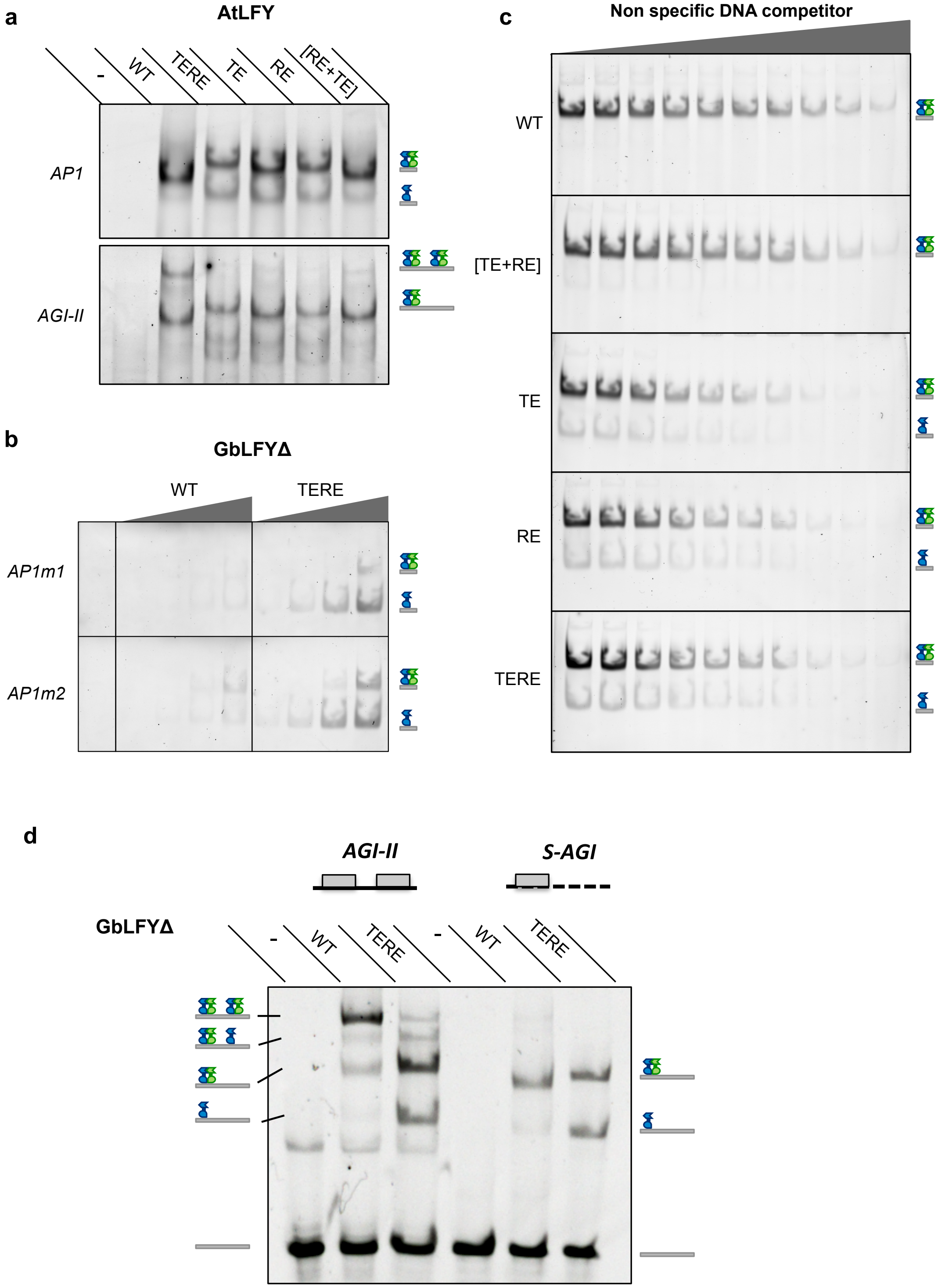

a
AtLFY
-
WT
TERE
TE
RE
[RE+TE]
AP1
AGI-II
b
GbLFYΔ
WT
TERE
AP1m1
AP1m2
c
Non specific DNA competitor
WT
[TE+RE]
TE
RE
TERE
d
AGI-II
S-AGI
GbLFYΔ
-
WT
TERE
-
WT
TERE

Supplementary Figure 4: Oligomerization through the SAM domain and *in vitro* DNA binding.

(a) EMSA with 10 nM *AP1* probe (top) or *AGI-II* probe (bottom) and 500 nM AtLFY, $AtLFY_{[RE+TE]}$, $AtLFY_{TE}$, $AtLFY_{RE}$ or $AtLFY_{TERE}$ proteins from *A. thaliana*.

(b) EMSA with 10 nM *AP1m1* or *AP1m2* probe (each carrying mutations on a half-site of the palindromic LFYbs) and 0, 50, 100, 250, 500 nM GbLFYΔ or $GbLFYΔ_{TERE}$ protein.

(c) Competition assay using EMSA. Binding reactions contained 10 nM *AP1* probe, 500 nM GbLFYΔ, $GbLFYΔ_{[RE+TE]}$,$GbLFYΔ_{TE}$, $GbLFYΔ_{RE}$ or $GbLFYΔ_{TERE}$ proteins and increasing concentration of non-specific unlabeled competitor DNA. Quantification of the fraction of bound DNA is shown in Figure 4d.

(d) EMSA illustrating the various complexes formed on 10 nM of a probe bearing one or two LFYbs from *AG* regulatory sequence (*S-AGI* and *AGI-II*, respectively) by 100 nM of either GbLFYΔ or $GbLFYΔ_{TERE}$. S-AGI is a synthetic sequence containing a single LFYbs (AGI).

For all EMSAs except in d, only the protein-DNA complexes are shown.

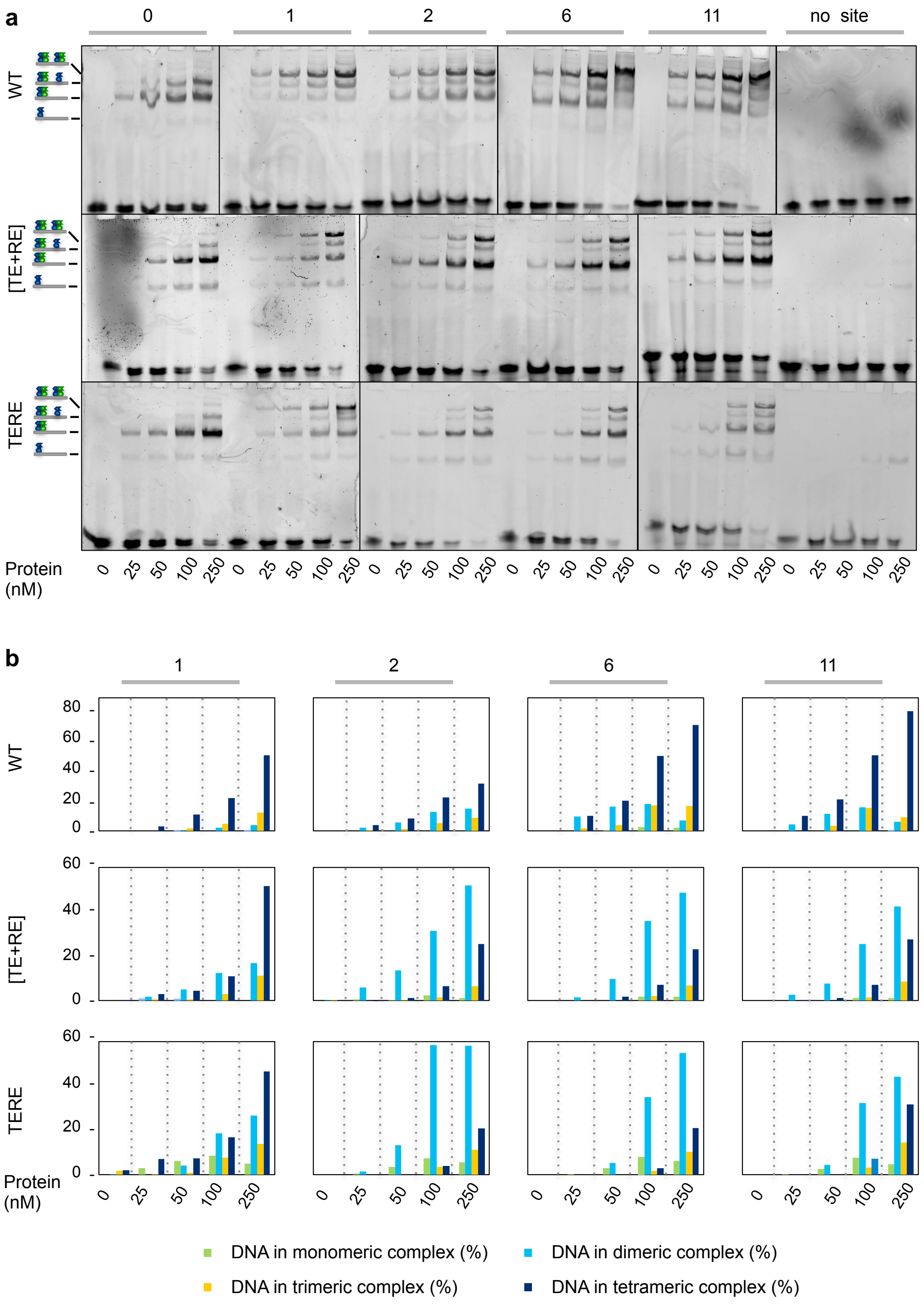

Supplementary Figure 5: *In vitro* DNA binding on synthetic DNA probes containing two LFY binding sites separated by variable distances.

(a) EMSA with 10 nM of a synthetic DNA probe containing two LFYbs spaced by 0, 1, 2, 6 or 11 bp and a range of GbLFYΔ, GbLFY$\Delta_{[RE+TE]}$ or GbLFYΔ_{TERE} protein concentrations. Only the protein-DNA complexes are shown. Schematic complexes are

shown to the left of gels. No tetrameric binding of GbLFYΔ tetramer with DNA was observed when the LFYbs are spaced by 0 bp.

(b) Quantification of the different protein/DNA complexes from (a). In contrast with GbLFY$\Delta_{[RE+TE]}$ or GbLFYΔ_{TERE} protein, the percentage of GbLFYΔ tetramer bound to DNA is always higher than the percentage of bound monomer, dimer or tetramer. At low protein concentrations (0-100 nM) of GbLFYΔ, the ratio tetramer/dimer is higher for sites spaced by 1 bp. With 0 bp spacing, the tetrameric complex is absent and was thus not quantified.

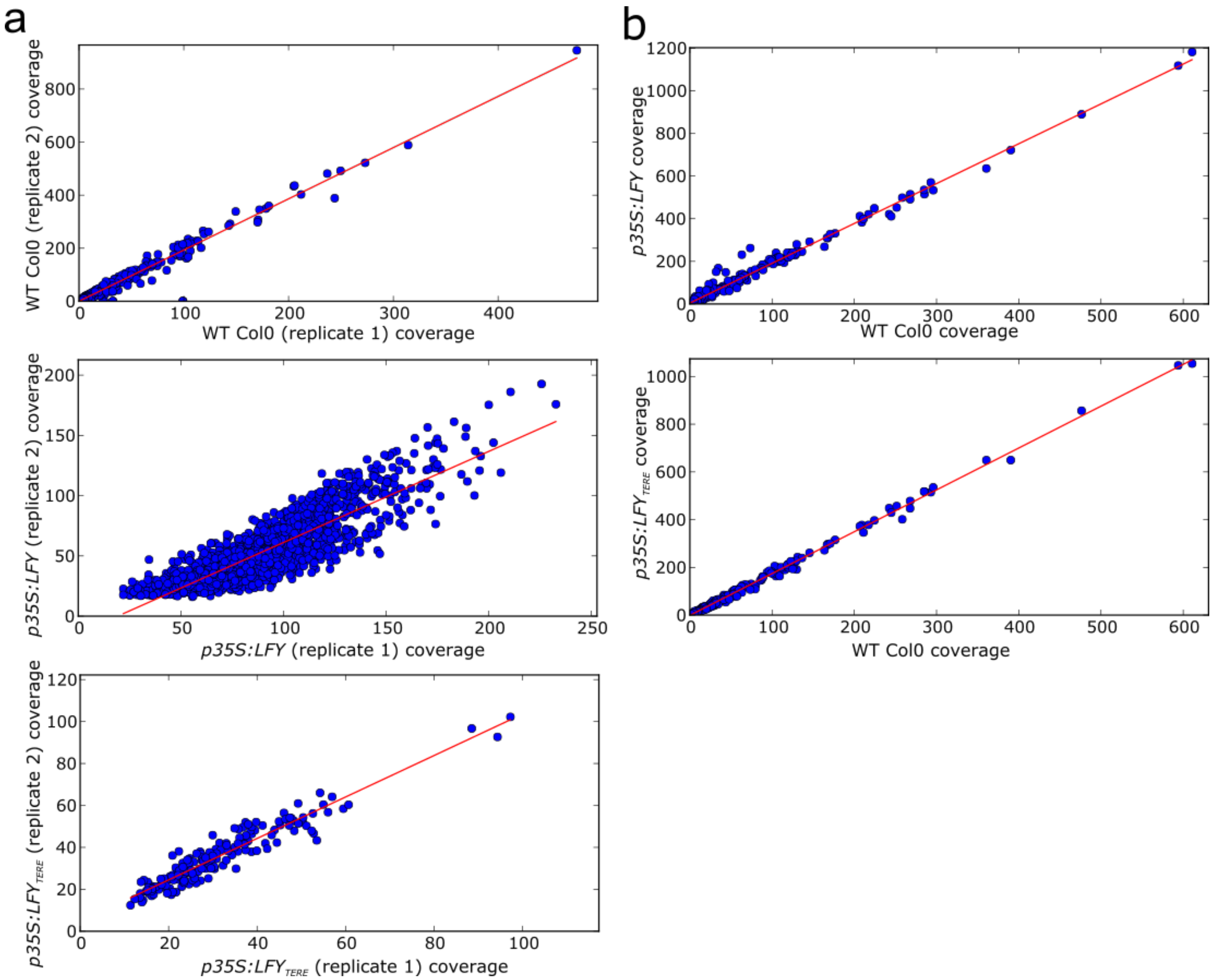

Supplementary Figure 6: ChIP-Seq normalization.

(a) Intra-genotype normalization: read coverages (in blue) and linear fit (in red) within genotypes for the background peaks (WT), the LFY bound regions (p35S:LFY) and the LFY_{TERE} bound regions ($p35S{:}LFY_{TERE}$).

(b) Inter-genotype normalization: background peaks detected in WT and present in all genotypes were used (see methods).

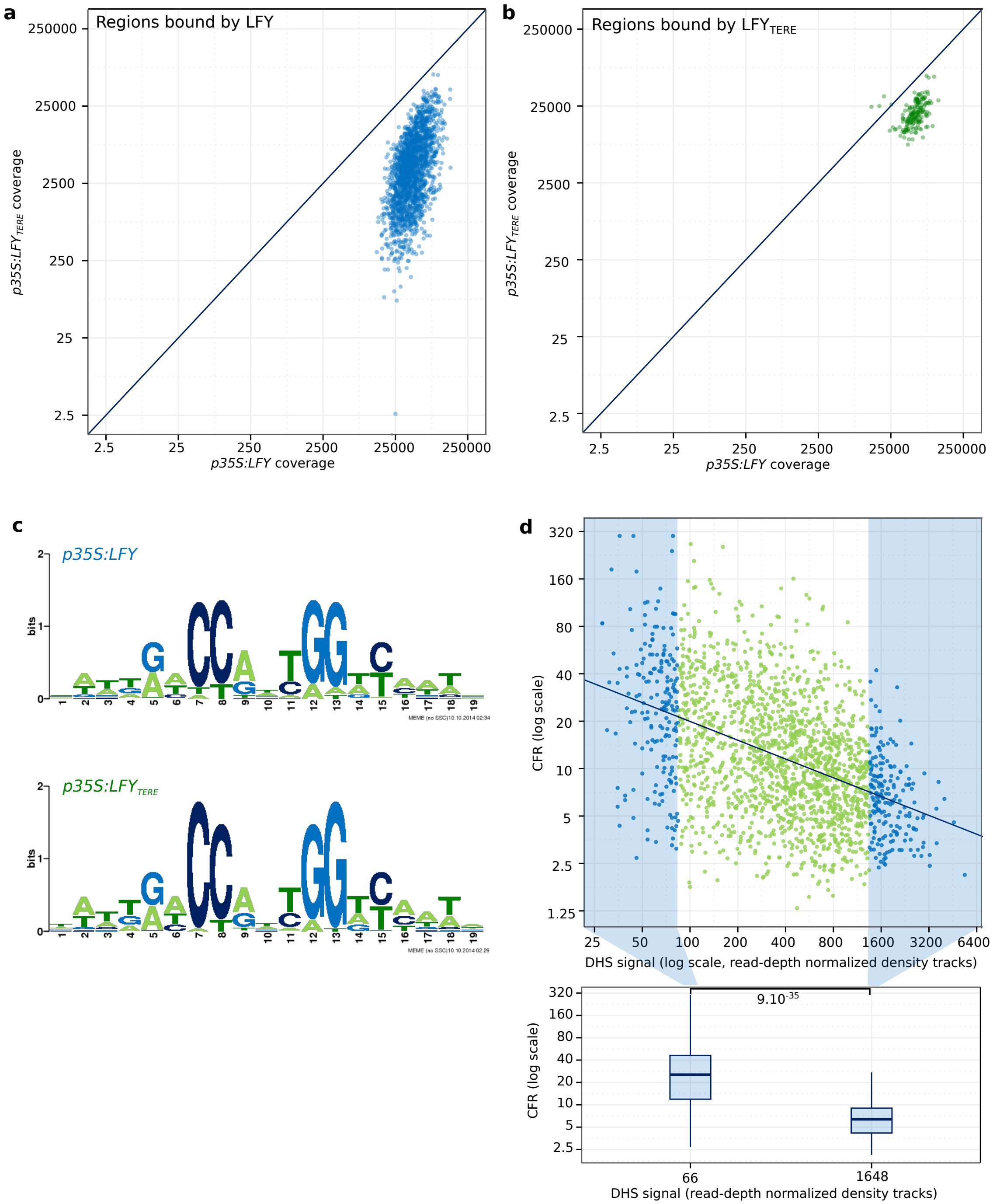

Supplementary Figure 7: Role of LFY-SAM oligomerization on the genome-wide LFY DNA binding *in planta*.

(a) Comparison of the coverages in *p35S:LFY* and *p35S:LFY$_{TERE}$* ChIP-Seq for the LFY-bound regions.

(b) Comparison of the coverages in *p35S:LFY* and *p35S:LFY$_{TERE}$* ChIP-Seq for the LFY$_{TERE}$-bound regions.

(c) Logos computed for overrepresented motifs identified with the MEME suite[3] in LFY (top) or LFY_{TERE} (bottom) bound regions. Symmetry was imposed.

(d) Same analysis as in Fig. 6 but performed on the dataset from 7 day-old seedlings[4]. Pearson's correlation test was performed between the CFR values and the DHS signal: r^2 = 19.80%, p-value = 1e-95. Data are shown in light blue and green, and linear fit in dark blue. The p-value for Mann-Whitney rank test on the two extreme groups, from pooling the 1954 p35S:LFY-bound regions in deciles, is indicated on the bottom graph.

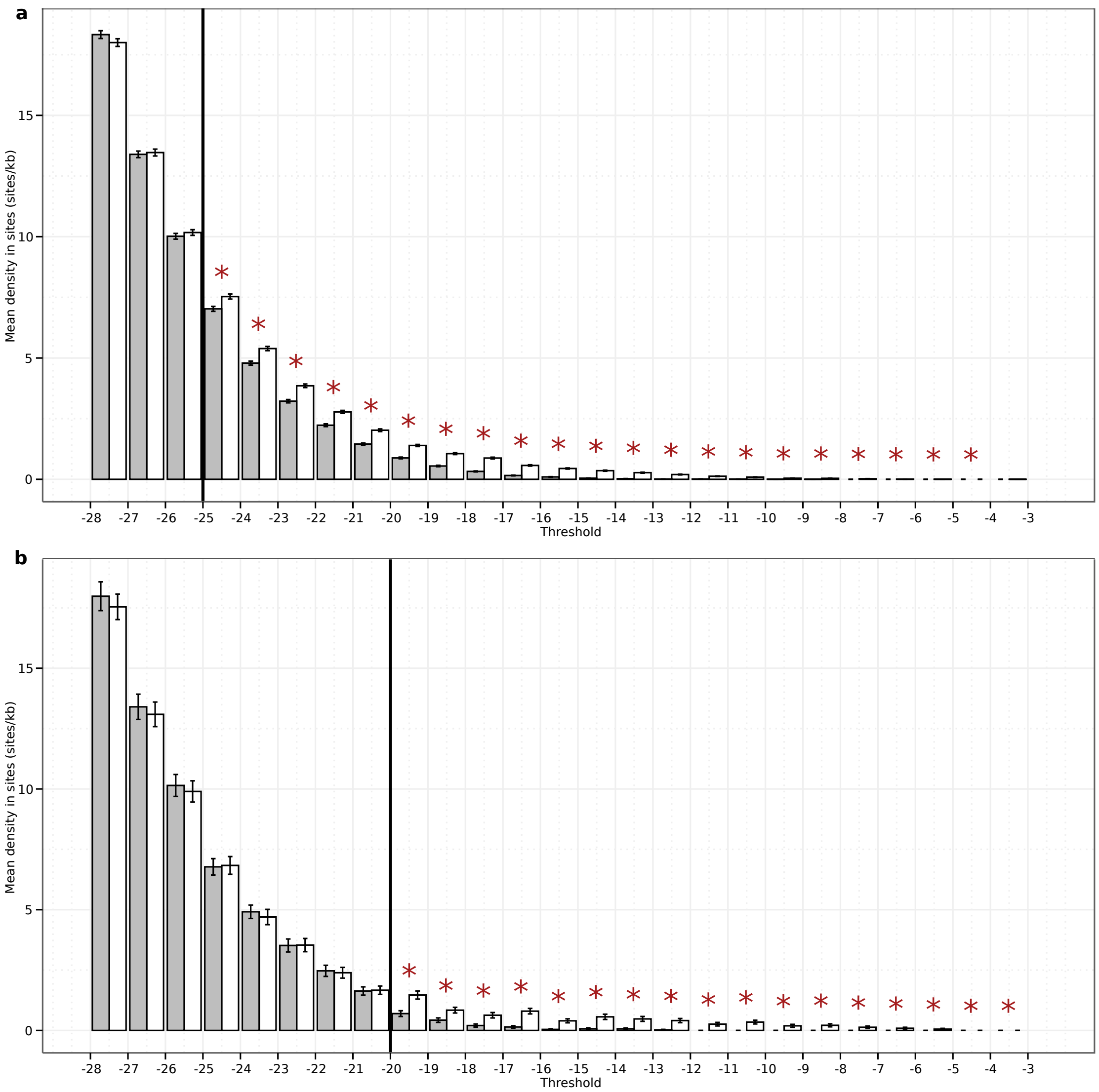

Supplementary Figure 8: Experimental determination of score threshold for the LFY DNA binding model.

(a) LFYbs densities in score windows for the bound regions in *p35S:LFY* (shown in white) and a negative control set (shown in grey). Red stars indicate a significantly higher density in the positive set as compared to the control according to Mann-Whitney rank test (p-value < 0.05). The black vertical line corresponds to the score threshold estimated by the program: -25

(b) Same as in (a) but performed on the LFY_{TERE}-bound regions. The score threshold is -20 in this case.

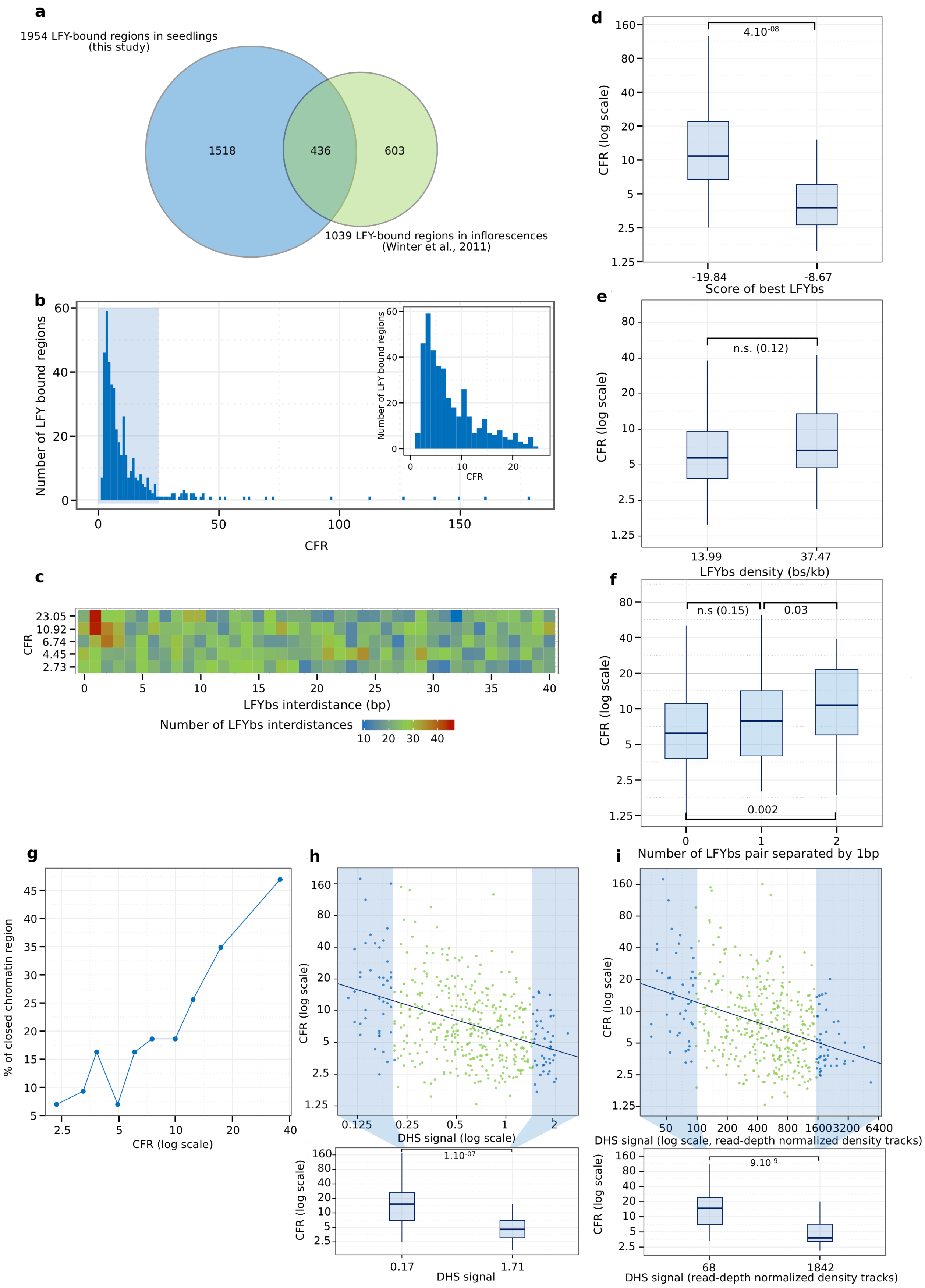

a
1954 LFY-bound regions in seedlings
(this study)
1518
436
603
1039 LFY-bound regions in inflorescences
(Winter et al., 2011)
b
Number of LFY bound regions
CFR
c
CFR
LFYbs interdistance (bp)
Number of LFYbs interdistances
d
4.10-08
CFR (log scale)
Score of best LFYbs
-19.84
-8.67
e
n.s. (0.12)
CFR (log scale)
LFYbs density (bs/kb)
13.99
37.47
f
n.s (0.15)
0.03
0.002
CFR (log scale)
Number of LFYbs pair separated by 1bp
g
% of closed chromatin region
CFR (log scale)
h
CFR (log scale)
DHS signal (log scale)
1.10-07
DHS signal
0.17
1.71
i
CFR (log scale)
DHS signal (log scale, read-depth normalized density tracks)
9.10-9
DHS signal (read-depth normalized density tracks)
68
1842

Supplementary Figure 9: Analyses of genomic regions bound by LFY both in *p35S:LFY* seedlings and wild-type inflorescences.

(a) Venn diagram showing the intersection between the 1954 regions bound in *p35S:LFY* seedlings in ChIP-Seq (this study) and 1039 bound regions in ChIP-Chip on wild-type inflorescence tissue [5,6]. The 436 regions found in the intersection are used in the analyses below.

(b) Histogram showing the distribution of CFR values for the 436 LFY bound regions.

(c) Heat map representing the distribution of distances between LFYbs. The 436 LFY bound regions in quintiles according to their CFR value (Y-axis shows the median CFR value for each quintile). X-axis shows the distance (in bp) separating two 19-bp LFYbs. Only distances smaller than 40 bp are shown.

(d) Boxplot showing the CFR of the LFY-bound regions with LFYbs of lowest and highest score (first and last deciles). X-axis shows the median LFYbs score value. P-value for Mann-Whitney rank test is indicated on the graph.

(e) Boxplot showing the CFR of the LFY-bound regions with lower and higher LFYbs density (first and last deciles of LFY bound regions). X-axis shows the median density value. The difference is not significant according to the P-value for Mann-Whitney rank test.

(f) Boxplot showing the CFR of regions depending on the number of LFYbs separated by 1 bp (zero, one and two pairs of LFYbs). P-values for Mann-Whitney rank test are indicated on the graph.

(g) Percentage of closed regions in deciles of the 436 LFY bound regions sorted according to their CFR.

(h) Comparison of the CFR of the 436 LFY-bound regions and the chromatin accessibility in 15-day-old seedlings (DHS signal)[7]. Pearson's correlation test was performed between the CFR and the DHS signal: $r^2 = 15\%$, p-value = 3.e-17. Data are shown in light blue and green and fitted values are shown in dark blue. Boxplot representing the CFR signal in the regions of highest and lowest DHS (first and last deciles, in light blue). P-value for Mann-Whitney rank test on the two extreme groups is indicated on the graph.

(i) Comparison of the CFR of the 436 LFY-bound regions and the chromatin accessibility

in 7-day-old seedlings (DHS signal)[4]. Pearson's correlation test was performed between the CFR and the DHS signal: r^2 = 14%, p-value = 1.e-15. Data are shown in light blue and green and fitted values are shown in dark blue. Boxplot representing the CFR signal in the regions of highest and lowest DHS (first and last deciles, in light blue). P-value for Mann-Whitney rank test on the two extreme groups is indicated on the graph.

CFR: coverage fold reduction, LFYbs: LFY binding site, n.s.: non significant

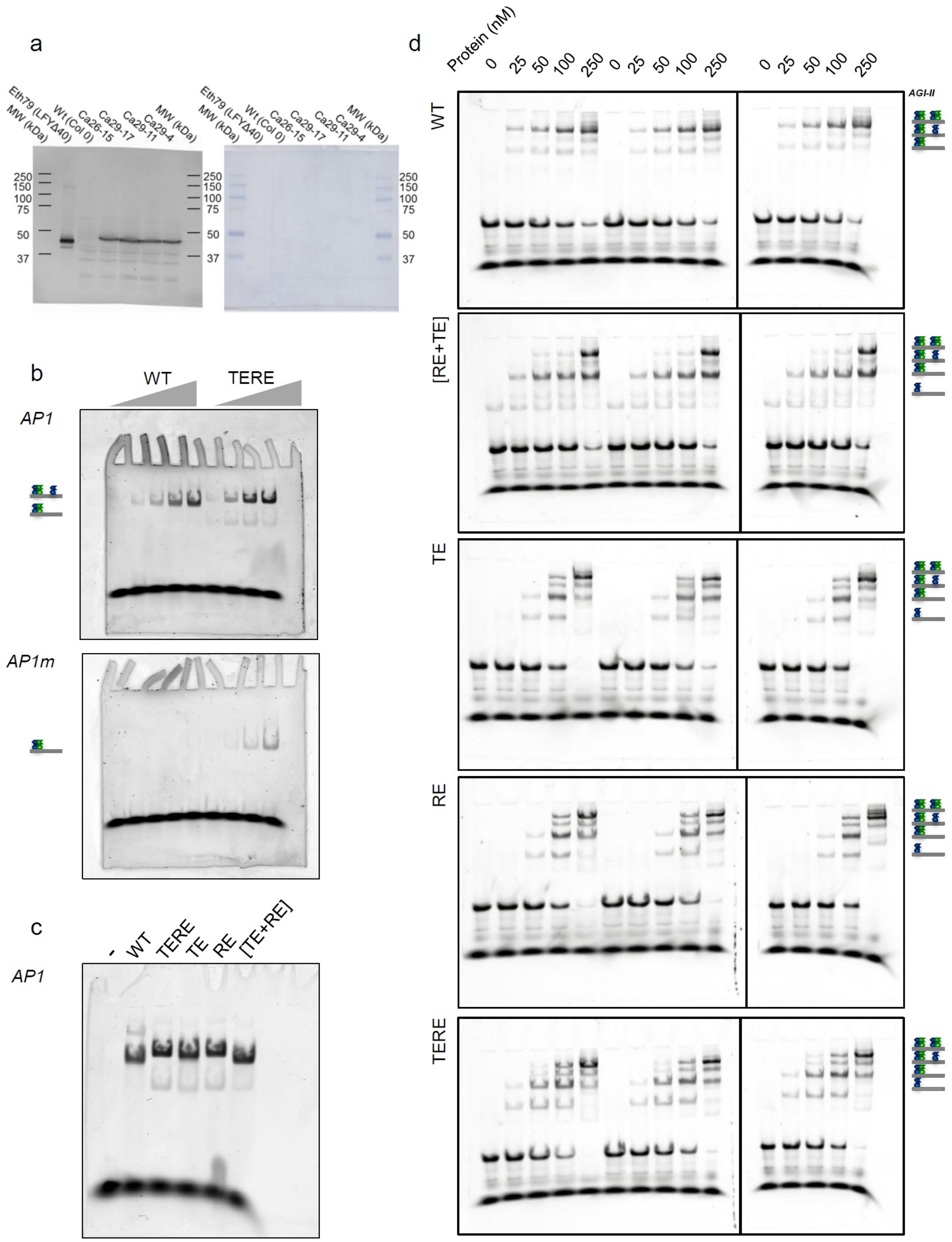
a
MW (kDa)
Eth79 (LFYΔ40)
Wt (Col 0)
Ca26-15
Ca29-17
Ca29-11
Ca29-4
MW (kDa)
250
150
100
75
50
37
b
WT
TERE
AP1
AP1m
c
-
WT
TERE
TE
RE
[TE+RE]
AP1
d
Protein (nM)
0
25
50
100
250
WT
[RE+TE]
TE
RE
TERE
AGI-II

Supplementary Figure 10: Uncropped Western Blot and EMSAs.

(a) Uncropped Western blot for LFY (left) corresponding to Supplementary Fig. 3a and scan of the blot showing the migration of protein standards (Precision plus protein standards, Bio-Rad) (right).

(b) Uncropped EMSAs corresponding to Fig. 4a.

(c) Uncropped EMSA corresponding to Fig. 4b.

(d) Triplicate of uncropped EMSAs corresponding to Fig. 4e.

Supplementary Tables

Supplementary Table 1: Molecular mass determination of GbLFY-SAM (WT and mutants, at different protein concentrations) and GbLFYΔ (WT and mutants, in complex with the DNA probe *AP1*).

Protein	Concentration ($mg.mL^{-1}$)	Molecular mass (kDa)	Number of monomers
**GbLFY-SAM**	1.6	78 (+/- 2.2)	6.1
	4.7	95 (+/- 1.6)	7.4
	5.8	97.3 (+/- 2.5)	7.6
	8.4	103.3 (+/- 2.1)	8.1
**GbLFY-SAM$_{TE}$**	4.6	12.5 (+/- 0.2)	1.0
**GbLFY-SAM$_{RE}$**	4.4	12.9 (+/- 0.2)	1.0
**GbLFY-SAM$_{TERE}$**	4.5	13.6 (+/- 0.8)	1.1
**GbLFY-SAM$_{[RE+TE]}$**	4.7	23 (+/- 1)	1.8
**GbLFYΔ-*AP1***	2.6	115.5 (+/- 5)	2.2
**GbLFYΔ$_{TE}$-*AP1***	2.6	98 (+/- 6)	1.8
**GbLFY$_{[RE+TE]}$-*AP1***	2.6	97.7 (+/- 4)	1.8

The molecular mass of GbLFY-SAM, GbLFY-SAM$_{TE}$, GbLFY-SAM$_{RE}$, GbLFY-SAM$_{TERE}$, GbLFYSAM$_{[RE+TE]}$ (equimolar mixture of GbLFY-SAM$_{TE}$ and GbLFY-SAM$_{RE}$), GbLFYΔ-*AP1*, GbLFYΔ$_{TE}$-*AP1* and GbLFYΔ$_{[RE+TE]}$-*AP1* was determined using SEC-MALLS (Fig. 2d and 4c). A volume of 50 μL was used for each protein. The protein concentration used, the measured molecular mass for each protein [+/- standard error], and the calculated number of protein molecules per complex are indicated. For determination of the molecular mass of the GbLFYΔ, GbLFYΔ$_{TE}$ and GbLFYΔ$_{[RE+TE]}$ in complex with the DNA probe *AP1*, 2.6 mg mL^{-1} (60 μM) protein and 15 μM DNA were used. The theoretical molecular masses for a GbLFY-SAM monomer, a GbLFYΔ monomer and *AP1* DNA are 12.8 kDa, 43.5 kDa and 18.4 kDa, respectively.

Supplementary Table 2: Complementation of a *lfy-12* null mutant by LFY and LFY_{TERE} expressed under the *LFY* promoter (*pLFY*)

Genotype		Rescue level			Total
		No	Weak	Strong	
pLFY:LFY	Number of plants	1	2	8	11
	% of plants	9.1	18.2	72.7	100
	Number of siliques / plant (median [max;min])	0	9.5 [8;11]	>20	
pLFY:LFY_{TERE}	Number of plants	1	13	3	17
	% of plants	5.9	76.5	17.6	100
	Number of siliques / plant (median [max;min])	0	3 [0;6]	>20	

Primary transformants (T1) obtained with the *pLFY:LFY* and *pLFY:LFY_{TERE}* constructs are distributed into 3 phenotypic classes. No rescue means that T1 plants show a *lfy-12* phenotype. A weak rescue means that plants produced either only sterile flowers, or first a low number of fertile flowers (scored by the number of siliques obtained after self-fertilization) and then a high number (>20) of sterile flowers lacking petals and stamens. A strong rescue means that plants produced fertile flowers (albeit with distorted petals). The distribution in phenotypic classes was shown to be significantly different by a Mann-Whitney rank test (p-value = 0.01, 99% p-value interval confidence [0.00922 - 0.01094]).

Supplementary Table 3: List of primers used for plasmid construction

Plasmid	Protein	Primers	
		Name	Sequence
**pETH164**	GbLFYΔ	oETH1067	GGCCATGGCAAAGGAATTGAGTTCTCTA
		oETH1068	CCCTCGAGTCAATGAGGTTCTTGGCTTTTCTC
**pETH195**	GbLFY-SAM	oETH1126	CCGGAACCTTCATCCATGGCAAGAAAGGAATTGAGTTCT
		oETH1128	ACTTGCTCGAGTCATGCCTGAGAGAGTGTGTCCAA
**pETH201**	AtLFY-SAM	oETH1130	TTAACCCCATGGCCCAGACGGCTGCTTTTGGGA
		oETH1131	CGTAACTCGAGCTATTGGGAGAGAGCATCAAGAGC
**pETH94**	AtLFY	oETH1031	TTCTCGAGGAAACGCAAGTCGTCGCCGCC
		oETH1032	TTAACATATGGATCCTGAAGGTTTCACGAGTGGC

Supplementary Table 4: List of primers used for mutagenesis

Primers used for mutagenesis				
Plasmid	Protein	Matrix vector	Primers for mutagenesis	
			Name	Sequence
**pCA21**	GbLFYΔ_{TE}	pETH164	oCA0019	TGGTTGAGATGGGTTTCACCGTCAATGAGTTGGTTAATATGACTGAACAAGAG
			oCA0020	CTCTTGTTCAGTCATATTAACCAACTCATTGACGGTGAAACCCATCTCAACCA
**pCA22**	GbLFYΔ_{RE}	pETH164	oCA0023	AGAAATATGGCATCAAGTCTGCAGTTGAGGCAGAAAAGAGAAGATTGGATGAG
			oCA0024	CTCATCCAATCTTCTCTTTTCTGCCTCAACTGCAGACTTGATGCCATATTTCT
**pCA39**	GbLFYΔ_{TERE}	pCA22	oCA0019	TGGTTGAGATGGGTTTCACCGTCAATGAGTTGGTTAATATGACTGAACAAGAG
			oCA0020	CTCTTGTTCAGTCATATTAACCAACTCATTGACGGTGAAACCCATCTCAACCA
**pCA23**	AtLFY$_{TE}$	pETH94	oCA0029	GTTAGGTTTTACGGCTAGCGAGCTTGTGGGTATGAAGGAC
			oCA0030	GTCCTTCATACCCACAAGCTCGCTAGCCGTAAAACCTAAC
**pCA24**	AtLFY$_{RE}$	pETH94	oCA0027	TACGGTATCAAAGCTGCCGTTGAGGCTGAACGGAGACGATTGC
			oCA0028	GCAATCGTCTCCGTTCAGCCTCAACGGCAGCTTTGATACCGTA
**pCA25**	AtLFY$_{TERE}$	pCA24	oCA0031	GTTAGGTTTTACGGCGAGCGAGCTCGTGGGTATGAAGGAC
			oCA0032	GTCCTTCATACCCACGAGCTCGCTCGCCGTAAAACCTAAC
**pCA15**	GbLFY-SAM$_{TE}$	pETH195	oCA0019	TGGTTGAGATGGGTTTCACCGTCAATGAGTTGGTTAATATGACTGAACAAGAG
			oCA0020	CTCTTGTTCAGTCATATTAACCAACTCATTGACGGTGAAACCCATCTCAACCA
**pCA17**	GbLFY-SAM$_{RE}$	pETH195	oCA0023	AGAAATATGGCATCAAGTCTGCAGTTGAGGCAGAAAAGAGAAGATTGGATGAG
			oCA0024	CTCATCCAATCTTCTCTTTTCTGCCTCAACTGCAGACTTGATGCCATATTTCT
**pCA20**	GbLFY-SAM$_{TERE}$	pCA17	oCA0019	TGGTTGAGATGGGTTTCACCGTCAATGAGTTGGTTAATATGACTGAACAAGAG
			oCA0020	CTCTTGTTCAGTCATATTAACCAACTCATTGACGGTGAAACCCATCTCAACCA

Supplementary Table 5: List of buffers used for protein purification

Buffer composition for protein purification		
**Buffer name**	**Proteins**	**Buffer composition**
**Buffer A**	GbLFY-SAM, GbLFY-SAM$_{TE}$, GbLFY-SAM$_{RE}$, GbLFY-SAM$_{TERE}$, AtLFY-SAM	20 mM Tris–HCl pH 8, 1 mM TCEP
**Buffer B**	GbLFYΔ, GbLFYΔ$_{TE}$, GbLFYΔ$_{RE}$, GbLFYΔ$_{TERE}$	1 M NaCl, 20 mM Tris–HCl pH 8.5, 1 mM TCEP
**Buffer C**	GbLFYΔ, GbLFYΔ$_{TE}$, GbLFYΔ$_{RE}$, GbLFYΔ$_{TERE}$ (SEC only)	0.8 M NaCl, 20 mM Tris–HCl pH 8.5, 1 mM TCEP
**Buffer D**	AtLFY, AtLFY$_{TE}$, AtLFY$_{RE}$, AtLFY$_{TERE}$	20 mM Tris–HCl pH 8.5, 1 mM TCEP

Supplementary Table 6: Oligonucleotides used for EMSA

Mutations are indicated in red, LFYbs in italics

Oligonucleotides used for EMSA assays		
Name	Sequence	Reference
AP1	GTTGGG*GAAGGACCAGTGGTCCGTA*CAATGT	LFY bs1 ; [8]
AP1m	GTTGGG*GAAGGAAAAGTAATCCGTA*CAATGT	LFY bs1 m3 ; [8]
AP1m1	GTTGGG*GAAGGAAAAGTGGTCCGTA*CAATGT	LFY bs1 m1 ; [8]
AP1m2	GTTGGG*GAAGGACCAGTAATCCGTA*CAATGT	LFY bs1 m2 ; [8]
AGI-II	CGTTTAAA*TTTAATCCAATGGTTACAA*TTTTAATACTATCA AATGTCTATTGGA*TTTATACCCAATGTGTTAAT*GGGTTG	[9]
S-AGI	TAAA*TTTAATCCAATGGTTACAA*CTATACATGTATGTATAA CTTGAATATGACTATGTTCTAGATTTGAAGTTGTTT	Synthetic probe

Supplementary Table 7: Mapped reads from ChIP-Seq

Genotype replicate	Mapped reads number
**Col_1**	893 004
**Col_2**	2 049 748
p35S:LFY$_{TERE}$_1	2 331 737
p35S:LFY$_{TERE}$_2	2 691 364
p35S:LFY_1	6 216 352
p35S:LFY_2	4 091 296

Supplementary Table 8: Coefficient used for ChIP-Seq normalization

Normalization step	Equation	Number of peaks	m	p-value	Correlation coefficient between samples (r)
**Col intra**	Col2 = m*Col1	521	1.93	3e-190	0.90
***p35S:LFY$_{TERE}$* intra**	*p35S:LFY$_{TERE}$*_2 = m**p35S:LFY$_{TERE}$*_1	176	0.99	1e-91	0.95
***p35S:LFY* intra**	*p35S:LFY_2* = m**p35S:LFY_1*	1954	0.76	0	0.87
***p35S:LFY$_{TERE}$* vs Col**	*p35S:LFY$_{TERE}$* = m*Col	355	1.75	0	1.00
***p35S:LFY* vs Col**	*p35S:LFY* = m*Col	355	1.87	0	0.99

Supplementary References

1. Ashkenazy, H., Erez, E., Martz, E., Pupko, T. & Ben-Tal, N. ConSurf 2010: calculating evolutionary conservation in sequence and structure of proteins and nucleic acids. *Nucleic Acids Res.* 38, W529–33 (2010).

2. Dumas, R., Joyard, J. & Douce, R. Purification and characterization of acetohydroxyacid reductoisomerase from spinach chloroplasts. *Biochem. J.* 262, 971–6 (1989).

3. Bailey, T. L. *et al.* MEME SUITE: tools for motif discovery and searching. *Nucleic Acids Res.* 37, W202–8 (2009).

4. Sullivan, A. M. *et al.* Mapping and Dynamics of Regulatory DNA and Transcription Factor Networks in A. thaliana. *Cell Rep.* 8, 2015–30 (2014).

5. Winter, C. M. *et al.* LEAFY target genes reveal floral regulatory logic, cis motifs, and a link to biotic stimulus response. *Dev. Cell* 20, 430–43 (2011).

6. Heyndrickx, K. S., de Velde, J. V., Wang, C., Weigel, D. & Vandepoele, K. A Functional and Evolutionary Perspective on Transcription Factor Binding in Arabidopsis thaliana. *Plant Cell* 26, 3894–3910 (2014).

7. Zhang, W., Zhang, T., Wu, Y. & Jiang, J. Genome-wide identification of regulatory DNA elements and protein-binding footprints using signatures of open chromatin in Arabidopsis. *Plant Cell* 24, 2719–31 (2012).

8. Benlloch, R. *et al.* Integrating long-day flowering signals: a LEAFY binding site is essential for proper photoperiodic activation of APETALA1. *Plant J.* 67, 1094–102 (2011).

9. Lohmann, J. U. *et al.* A molecular link between stem cell regulation and floral patterning in Arabidopsis. *Cell* 105, 793–803 (2001).
